# Maternal invasion history of *Aedes aegypti* and *Aedes albopictus* into the Isthmus of Panama: Implications for the control of emergent viral disease agents

**DOI:** 10.1371/journal.pone.0194874

**Published:** 2018-03-26

**Authors:** Gilberto A. Eskildsen, Jose R. Rovira, Octavio Smith, Matthew J. Miller, Kelly L. Bennett, W. Owen McMillan, Jose Loaiza

**Affiliations:** 1 Instituto de Investigaciones Científicas y Servicios de Alta Tecnología, Ciudad del Saber, Apartado, Panamá, República de Panamá; 2 Department of Biotechnology, Acharya Nagarjuna University, Guntur, India; 3 Smithsonian Tropical Research Institute, Balboa Ancon, Unit 0948, Panama, Republic of Panama; 4 Centro del Agua del Trópico Húmedo para América Latina y el Caribe, Panamá, República de Panamá; 5 Sam Noble Oklahoma Museum of Natural History and Department of Biology, University of Oklahoma, Norman, OK, United States of America; 6 Programa Centroamericano de Maestría en Entomología, Universidad de Panamá, Panamá, República de Panamá; Universita degli Studi di Pavia, ITALY

## Abstract

Despite an increase in dengue outbreaks and the arrival of chikungunya and Zika disease in Panama, studies on the demographic history of the invasive *Aedes* mosquitoes that are the principle vectors of these diseases are still lacking in this region. Here, we assess the genetic diversity of these mosquitoes in order to decipher their invasion histories into the Isthmus of Panama. DNA sequences from the mitochondrial cytochrome C oxidase I gene obtained from 30 localities in 10 provinces confirmed the presence of more than one mitochondrial haplogroup (i.e., maternal lineage) in each species. The invasion of *Aedes albopictus* was likely from temperate European countries, as the most frequent and widespread haplogroup in Panama harbored variants that are uncommon elsewhere in the Americas. Two infrequent and geographically restricted *Ae*. *albopictus* haplotypes appear to have subsequently invaded Panama from neighboring Costa Rica and the USA, respectively. In addition, we recovered two deeply divergent mitochondrial clades in Panamanian *Aedes aegypti*. The geographic origins of these clades is unknown, given that divergence in the mitochondrial genome is probably due to ancient population processes within the native range of *Ae*. *aegypti*, rather than due to its global expansion out of Africa. However, Panamanian *Ae*. *aegypti* mitochondrial sequences within the first clade were closely related to others from Colombia, Bolivia, Brazil, Mexico and the USA, suggesting two separate invasions from Western Hemisphere source populations. The pattern of increased genetic diversity in *Aedes* mosquitoes in Panama is likely facilitated by the numerous land and water inter-connections across the country, which allows them to enter via sea- and land-transportation from Europe, North, Central and South America. Our results here should be considered in disease mitigation programs if emergent arboviruses are to be effectively diminished in Panama through vector suppression.

## Introduction

*Aedes aegypti* (Linnaeus 1762) and *Aedes albopictus* (Skuse 1894) are two mosquitoes with the ability to invade and colonize human-altered ecological niches. These mosquitoes can transmit several arboviruses (e.g., arthropod-borne viruses) including Yellow Fever (YF) and dengue (DENG) [1,2]. In addition, newly emerging viral pathogens affecting human populations such as Chikungunya (CHIKV) and Zika (ZIKV) have expanded worldwide and successfully established in new geographical areas due to the intercontinental biological invasion of these mosquitoes [3–5]. *Ae*. *aegypti* is native to Africa while *Ae*. *albopictus* originates from Asia; both species now have a worldwide distribution. The global expansion of the YF mosquito, *Ae*. *aegypti*, began earlier than that of *Ae*. *albopictus*. The former species likely invaded the Americas through inter-continental ship cargo from Africa, including the slave trade, roughly 350 years ago [6,7]. In contrast, the Asian tiger mosquito, *Ae*. *albopictus*, invaded the Americas more recently, around 1980, likely due to the importation of goods by sea, and almost simultaneously initiated a rapid demographic expansion across Africa, the Middle East and Europe, which continues even today [8–10].

The ability of *Aedes* mosquitoes to expand their geographic ranges has been linked to the global increase in international travel, and in particular, to the development of an international market in used tires [4], as demonstrated by the invasion of *Ae*. *albopictus* into the Americas, Europe and Africa [8–12]. The first detection of *Ae*. *albopictus* in Continental USA occurred in Texas, in 1985 [13,14]. Shortly after that, *Ae*. *albopictus* was found for the first time in Brazil and also in South Africa, via imported tires from Japan [15–17]. Although there is less evidence for human-assisted movement of *Ae*. *aegypti* through the shipment of used tires, a population of this species was introduced into the Netherlands in 2010, via a tire shipment originally from Miami, confirming that both mosquito species are notable human hitchhikers [8,12,18,19].

*Aedes aegypti* was first reported in Panama in 1912, during the construction of the Panama Canal, and coincident with major YF epidemics. By 1950, the vector control program of the Pan-American Health Organization (PAHO) likely eradicated *Ae*. *aegypti* from most of the Americas, including Panama “via aggressive DDT spraying”. However, this species became re-established subsequently, most likely between 1969 and 1985, and it is once again widespread [20]. Prior studies of the demographic history of *Ae*. *aegypti* from the Americas reported the co-existence of two divergent mitochondrial DNA (mtDNA) clades, which supports the hypothesis of multiple introductions, supposedly from western and eastern Africa, respectively [18] [19,21,22]. Alternatively, the co-occurrence of these clades in American *Ae*. *aegypti* populations could be due to relic African-originated strains that survived PAHO eradication plans in 1950, along with subsequent re-infestations from non-African source populations [23–25]. However, more recent studies posit that deep divergence in the mitochondrial genome of *Ae*. *aegypti* from around the world might be an artifact of more ancient population processes (i.e., retained ancestral polymorphisms) within its native range in Africa, rather than due to signatures of its global expansion [26,27]. The last view further implies that prior inferences about the invasion history of *Ae*. *aegypti* into the Americas may be mistaken as these clades do not necessary represent two separate introductions.

On the other hand, *Ae*. *albopictus* was first collected in Panama in 2004, and is estimated to have invaded more than 60% of the country during the 7 years after its initial introduction. The movement of human goods, likely used tires, seems to promote the rapid colonization process of *Ae*. *albopictus* in Panama. Specifically, its spatial movement across the country is best explained by the road network system as opposed to other factors such as climate, population density, landscape use or in combination [28]. Prior population genetic studies based on allozymes and mitochondrial DNA suggest that *Ae*. *albopictus* invaded the Americas multiple times, perhaps from both temperate and tropical source Asian populations [14,29]. Based on patterns of population polymorphism in this vector using the mitochondrial NADH dehydrogenase subunit five gene (ND5) and the cytochrome C oxidase subunit one gene (CO1), several studies suggest that early colonization of the USA was achieved by temperate strains from Japan, whereas the initial invasion of Brazil was likely accomplished by tropical strains from Southeast Asia [8,9,29–34]. Indochina being the most probable source population [35,36]

In Panama, details about the invasion history of *Aedes* mosquitoes are still limited. A recent study by Futami and collaborators [37] suggested that *Ae*. *albopictus* invaded Panama on one single occasion via land transportation from Costa Rica, but the low number of mosquitoes and few sites sampled in that study make any conclusion incomplete. *Ae*. *albopictus* was first collected in Panama City, in 2004, from where it appears to have expanded westward along the Pan-American Highway. However, the lack of confirmed samples from parts of Veraguas, Cocle and its complete absence from the Azuero Peninsula raises the possibility that its occurrence in Chiriqui, near the Costa Rican border, is the result of a separate introduction from Costa Rica [28].

Here, we assess the levels of genetic diversity in *Ae*. *aegypti* and *Ae*. *albopictus*, two primary vectors of emergent arboviruses in Panama, in an effort to decipher their invasion histories. In so doing, we seek to gain information about the following points: (1) the number of invading maternal lineages of each species into Panama and (2) the geographic origin of genetic diversity within species and its geographic distribution across the Isthmus, using partial sequences of the mtDNA *CO1* and comparable samples of both taxa from the whole country. In addition, we compare the invasion history of these two mosquitoes and examine the implications of our findings for vector control strategies and for the transmission of emergent arboviruses in Panama. We expected to find greater genetic diversity in populations of *Ae*. *aegypti* due to an older time of Panama invasion relative to the 2004 invasion of *Ae*. *albopictus*. Also, due to the geographic position of the Isthmus of Panama, with cargo ship traffic from around the world and through the Panama Canal, along with its land connection to both North and South America, we expected to find signatures of multiple invading maternal lineages in both species. Explicitly, this hypothesis predicts mixed continental origins among populations of Panamanian *Aedes* species.

## Materials and methods

### Mosquito sampling

**Fig 1:**
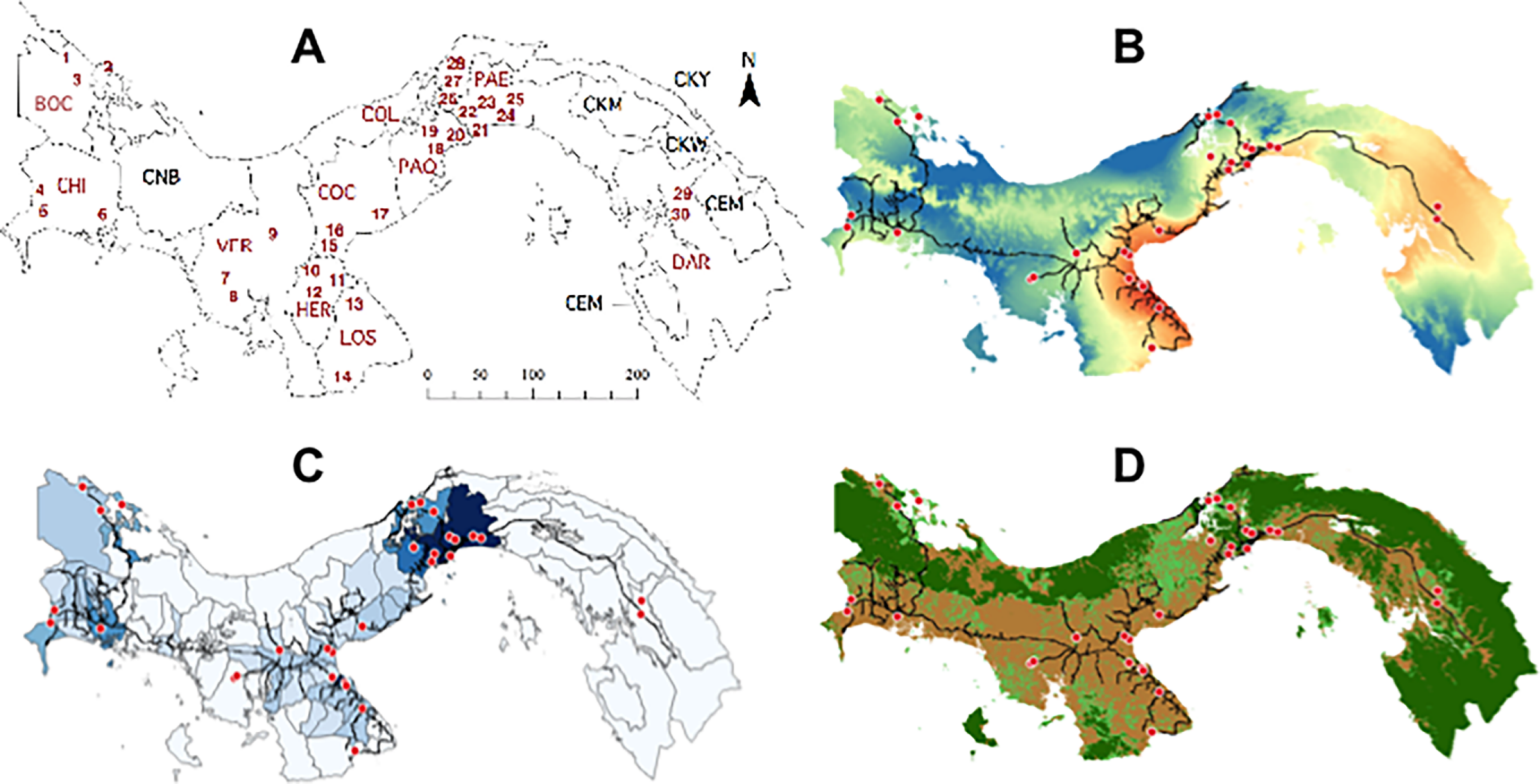
**Map (A) depicts *Aedes* collection localities from Panama**. Localities (numbers in red), provinces (codes in red), and *comarcas* (codes in black; a *comarca* is an indigenous political region). Each province and comarca is labeled. BOC = Bocas del Toro; CHI = Chiriquí, CNB = Comarca Ngobe-Buglé, VER = Veraguas; HER = Herrera; LOS = Los Santos; COC = Coclé, COL = Colón; PAE = Panamá Este; PAO = Panamá Oeste; CKY = Comarca Kuna Yala; CKM = Comarca Kuna de Madungandí; CKW = Comarca Kuna de Wargandí, CEM = Comarca Embera Wounaan; DAR = Darién. CKM is a territory within PAN province; CKW is a territory within DAR province. **Maps (B), (C) and (D)** depict collection site (circles in red) in relation to Precipitation, Population Density and Landscape use in Panamá, respectively. The dark lines in maps B, C and D represent the main roads across the country.

Our sampling procedure follows the Panamanian Ministry of Health (MINSA) Vector Control Department protocols for collecting immature (i.e., eggs, larvae, and pupae) and adult mosquitoes. In brief, randomly chosen houses, at a distance of 2 kilometers from each other, were checked for the presence of resting adult mosquitoes and developing immature life stages in water filled containers. Only one individual mosquito per species was selected from each house, in an attempt to avoid picking siblings from the same house. Geographic coordinates for sampling points were recorded using a hand-held Global Positioning System (GPS) unit (Garmin International, Olathe, KS), set to the WGS84 datum, and imported into ArcView GIS software (Environmental Systems Research Institute, Redlands, CA) to create maps of sample distribution in relation to precipitation, human population density and landscape use (Fig 1). Updated GIS data layers were obtained from the Centro del Agua del Trópico Humedo para America Latina y el Caribe (CATHALAC), in Panama City. Mosquitoes were transported to the laboratory at the Instituto de Investigaciones Científicas y Servicios de Alta Tecnología (INDICASAT-AIP) and identified to species level using a taxonomic key [38]. Immature stages of mosquitoes were reared to adulthood in the insectary under standardize conditions (e.g., LD 12:12 hours, 85% relative humidity, and 37°C) and kept separately in the lab at -80°C until molecular procedures. Both adults and larvae were stored in absolute ethanol at– 80° C until DNA extraction. *Aedes aegypti* and *Ae*. *albopictus* were sampled from a total of 30 localities from 10 different provinces or geopolitical territories of Panama, between August 2014 and November 2015 (Table 1; Fig 1).

**Table 1:** Localities, Counties and Provinces where *Aedes* mosquitoes were sampled in Panama, plus their geographic coordinates and relative species numbers.

Locality	County	Province (Code)	Latitude	Altitude	*Ae*. *aegypti*	*Ae*. *albopictus*
1. Guabito	Bocas del Toro	Bocas del Toro (BOC)	9° 28.39' N	- 82° 34.12' W	4	0
2. Isla Colon	Isla Colon		9° 20.32' N	- 82° 14.54' W	2	0
3. Nuevo Almirante	Almirante		9° 17.51' N	- 82° 25.12' W	4	0
4. Aserrio	Aserrio	Chiriquí (CHI)	8° 31.38' N	- 82° 47.57' W	7	5
5. El progreso	Barú		8° 25.47' N	- 82° 49.54' W	0	5
6. San José	Pedregal		8° 23.07' N	- 82° 25.30' W	8	4
7. San José	Soná	Veraguas (VER)	8° 00.20' N	- 81° 19.32' W	6	4
8. San Pablo,	Soná		8° 01.05' N	- 81° 18.05' W	0	4
9. San Francisco	Canto del Llano		8° 13.08' N	- 80° 57.13' W	6	5
10. Parita	Parita	Herrera (HER)	8° 00.32' N	- 80° 31.06' W	6	6
11. San Juan Bautista	Chitré		7° 57.54' N	- 80° 24.47' W	5	6
12. La Villa	La Villa	Los Santos (LOS)	7° 56.26' N	- 80° 24.17' W	5	3
13. Macaracas	Los Santos		7° 26.07' N	- 80° 19.51' W	3	6
14. Las Tablas	Las Tablas		7° 45.56' N	- 80° 16.16' W	3	3
15. Barrios unidos	Barrios unidos	Coclé (COC)	8° 12.07' N	- 80° 30.57' W	4	0
16. Aguadulce	Aguadulce		8° 13.48' N	- 80° 33.18' W	4	7
17. Antón	Antón		8° 24.06' N	- 80° 16.16' W	4	6
18. Burunga	Burunga	Panama Oeste (PAO)	8° 57.55' N	- 79° 40.32' W	4	4
19. Los Cerezos 2	Vacamonte		8° 54.02' N	- 79° 42.06' W	4	3
20. Nuevo Emperador	Chorrera		9° 00.38' N	- 79° 50.58' W	5	5
21. Las Garzas	Pacora	Panama Este (PAE)	9° 05.00' N	- 79° 17.26' W	4	4
22. Chorrillo	Ancón		8° 56.57' N	- 79° 32.44' W	2	3
23. Camino Omar	24 De Diciembre		9° 06.06' N	- 79° 21.37' W	2	4
24. San Pablo	Las Cumbres		9° 05.40' N	- 79° 32.56' W	3	3
25. El Valle	Omar torrijos		9° 04.23' N	- 79° 30.33' W	4	3
26. Buena Vista	Buena Vista	Colón (COL)	9° 17.14' N	- 79° 40.55' W	3	3
27. Puerto pilón	Puerto pilón		9° 21.40' N	- 79° 47.31' W	4	4
28. Villa del Caribe	Cristobal		9° 20.39' N	- 79° 51.42' W	6	5
29. Bello horizonte	Metetí	Darién (DAR)	8° 39.09' N	- 77° 58.26' W	6	7
30. Piedra candela	Metetí		8° 29.53' N	- 77° 58.41' W	4	5

Details about each locality and province can be found in Fig 1 and in S1 Fig. The last two columns represent the number of *Ae*. *aegypti* and/or *Ae*. *albopictus* mosquitoes used in the phylogeographic analysis. (-) Indicates that zero individuals were sampled for the respective species, in that particular locality.

### DNA extraction, PCR and sequencing procedures

DNA was extracted from whole insect bodies using DNAeasy Blood & Tissue kit (QIAgen®, Hilden, Germany) following the manufacturer’s recommendations. We intended to amplify fragments of 860 (*Ae*. *aegypti*) and 550 (*Ae*. *albopictus*) base-pairs from the mtDNA *CO1* gene using primers published by Paupy *et al*. [22] for *Ae*. *aegypti* and Kamgang *et al*. [39] for *Ae*. *albopictus*. PCR reactions were performed in a MaxyGene™ Thermal Cycler (Axygene, USA) in a 30 ul volume reaction mixture. Thermal conditions are published elsewhere [9,22]. PCR products were checked via agarose gel electrophoresis in Tris-borate-EDTA buffer, stained with 0.5 mg/ml ethidium bromide and visualized on UV light. Subsequently, amplicons were purified using ExoSAP-it (USB Corporation, Cleveland, USA) [40], and sent for Sanger sequencing to the Macrogen Sequencing Service, Seoul, Korea (http://www.macrogen.com/eng/). Forward and reversed sequences were edited and aligned in Geneious v 7.1 (http://www.geneious.com/) [41] using Muscle and Clustal algorithms. The GenBank accession numbers of our sequences are KX171382—KX171402.

### Data analysis

We calculated Tajima's *D* [42] and the mismatch distribution (MD) [43] in DnaSP v.5.0 [44] to test for signals of demographic expansion as possible factors shaping the historical demography of *Ae*. *aegypti* and *Ae*. *albopictus*. We tested the fit of the MD for each mosquito species to a model of population expansion, as this trend is anticipated when arthropods invade novel habitats/geographic areas and adapt to distinct ecological conditions. Furthermore, we examined intra-species evolutionary relationships using a series of phylogenetic network approaches in PopART (http://popart.otago.ac.nz/). The Minimum Spanning (MS), Median Joining (MJ), Tight Span Walker (TSW), Integer NJ Net (INJN) and Parsimony (TCS) aim at portraying the most likely reticulating pattern among unique molecular variants (e.g., haplotypes). However, they start from different assumptions given the data (http://popart.otago.ac.nz/). We also calculated the average number of pairwise differences among *CO1* sequences for *Ae*. *aegypti* and *Ae*. *albopictus* and for the haplogroups (e.g., invading maternal lineages) discovered within each species, and estimated their nucleotide divergence using DnaSP v.5.0[44].

We also estimated intra-species metrics of genetic diversity including the number of polymorphic sites, haplotype diversity, and nucleotide diversity for 10 *Ae*. *aegypti* and 9 *Ae*. *albopictus* populations representing different Panamanian provinces (Table 2; Fig 1). We performed Neighbor-Joining (NJ) phylogenetic analyses in MEGA v.6.0 [45]to assess the relationships between Panamanian haplotypes of *Aedes* mosquitoes and worldwide samples of both species. Here, we combined the *CO1* haplotypes in our data sets with additional sequences from GenBank (http://blast.ncbi.nlm.nih.gov/) encompassing *CO1* haplotypes of *Ae*. *aegypti* (S2 Table) and *Ae*. *albopictus* (S3 Table) from Africa, Asia, North-Central and South America as well as from the Caribbean.

**Table 2:** Intra-population diversity metrics for *Ae*. *aegypti* and *Ae*. *albopictus* from 10 Provinces of Panama, based on analyses with molecular sequences of the *CO1* gene.

	***Aedes aegypti***			***Aedes albopictus***		
Province	S	Hd	π	S	Hd	π
BOC	13	0.467	0.008	-	-	-
CHI	13	0.562	0.009	6	0.846	0.004
VER	16	0.773	0.011	4	0.718	0.004
HER	13	0.033	0.006	2	0.030	0.001
LOS	16	0.745	0.011	2	0.030	0.001
COC	13	0.439	0.006	6	0.718	0.004
PAO	22	0.885	0.011	7	0.818	0.050
PAE	22	0.838	0.009	8	0.778	0.004
COL	19	0.910	0.011	7	0.742	0.006
DAR	18	0.778	0.011	5	0.773	0.004
**Total**	**28**	**0.766**	0.010*	**9**	**0.819**	0.005*

Intra-population diversity metrics are given for both species and per geographic area or province by S = number of segregating sites; Hd = haplotype diversity; π = nucleotide diversity in columns 3, 4 and 5, respectively. Details about sampling localities and provinces can be found in Fig 1, Table 1 and in S1 Fig. The (-) symbol indicates that zero individuals were analyzed in that particular locality.

P value for the comparison of nucleotide diversity (π) between Ae. aegypti and Ae. albopictus using a two-tailed Mann Whitney test was P = 0.0012* (statistically significant). P value for the comparison of haplotype diversity (Hd) between these species using a two-tailed Mann Whitney test was P = 0.795 (not statistically significant).

## Results

We generated 239 mtDNA *CO1* sequences (122 individuals comprising full overlap of 728 base pairs from *Ae*. *aegypti* and 117 individuals comprising full overlap of 461 bp from *Ae*. *albopictus*) from 245 individuals attempted (97% amplification success rate). We recovered 13 unique haplotypes in *Ae*. *aegypti* and a total of 28 polymorphic sites (out of 728 bases analyzed), while in *Ae*. *albopictus* we recovered 8 unique haplotypes and 9 polymorphic sites (out of 461 bases analyzed) (Table 2). Estimated nucleotide diversity was higher in *Ae*. *aegypti* than in *Ae*. *albopictus* (*π* = 0.0096 *vs π* = 0.0048), but haplotype diversity was higher in *Ae*. *albopictus* (*Hd* = 0.819, SD = 0.33) than in *Ae*. *aegypti* (*Hd* = 0.766, SD = 0.27) (Table 2). Outcomes from a Mann Whitney test indicate that nucleotide diversity differences between *Ae*. *aegypti* and *Ae*. *albopictus* are statistically significant (U = 12, p < 0.05 two-tailed), while differences in haplotype diversity between species are not (U = 51, p < 0.05 two-tailed) (Table 2).

**Fig 2:**
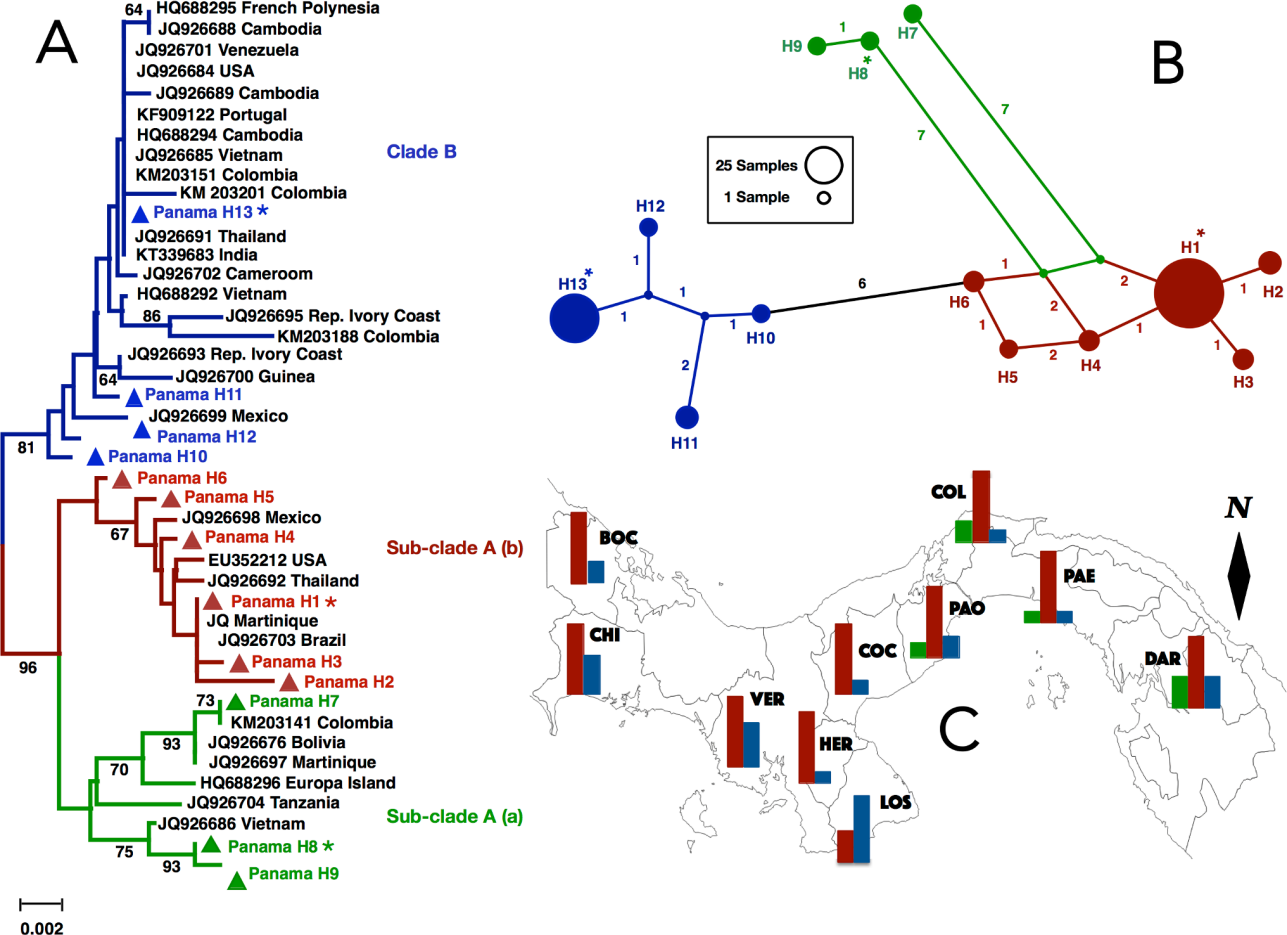
**(A) Neighbor-joining phylogenetic tree of Panamanian and worldwide CO1 haplotypes of *Aedes aegypti* from GenBank** (http://blast.ncbi.nlm.nih.gov/). Panamanian haplotypes belonging to sub-Clade A (a), sub-Clade A (b) and Clade B are shown in green, red and blue triangles, respectively. Bootstrap values depicting branch support higher than 60% are shown in the tree. Asterisks (*) in Haplotype 1 and Haplotype 13 indicate most frequent Panamanian haplotypes within sub-Clade A (b) and Clade B, respectively. **(B) TCS network depicting mutational relationships among three CO1 haplogroups of *Aedes aegypti*.** Haplogroups 1, 2 and 3 mimic the color of sub-Clade A (b), Clade B and sub-Clade A (a), in that order. Haplotypes are represented by circles and their sizes reflect their population frequencies. Missing haplotypes are represented by blue and green dots and numbers along lines are mutational differences. Haplogroups 1, 2 and 3 match those in Table 3. **(C) Geographic distribution of haplogroups 1 (red), 2 (blue) and 3 (green) across Panama.** Bars correspond to the regional frequency of that haplogroup per sampling Province (see Table 1 for additional details). Black diamond (i.e., North arrow) indicates the direction to the geographic North Pole.

**Fig 3:**
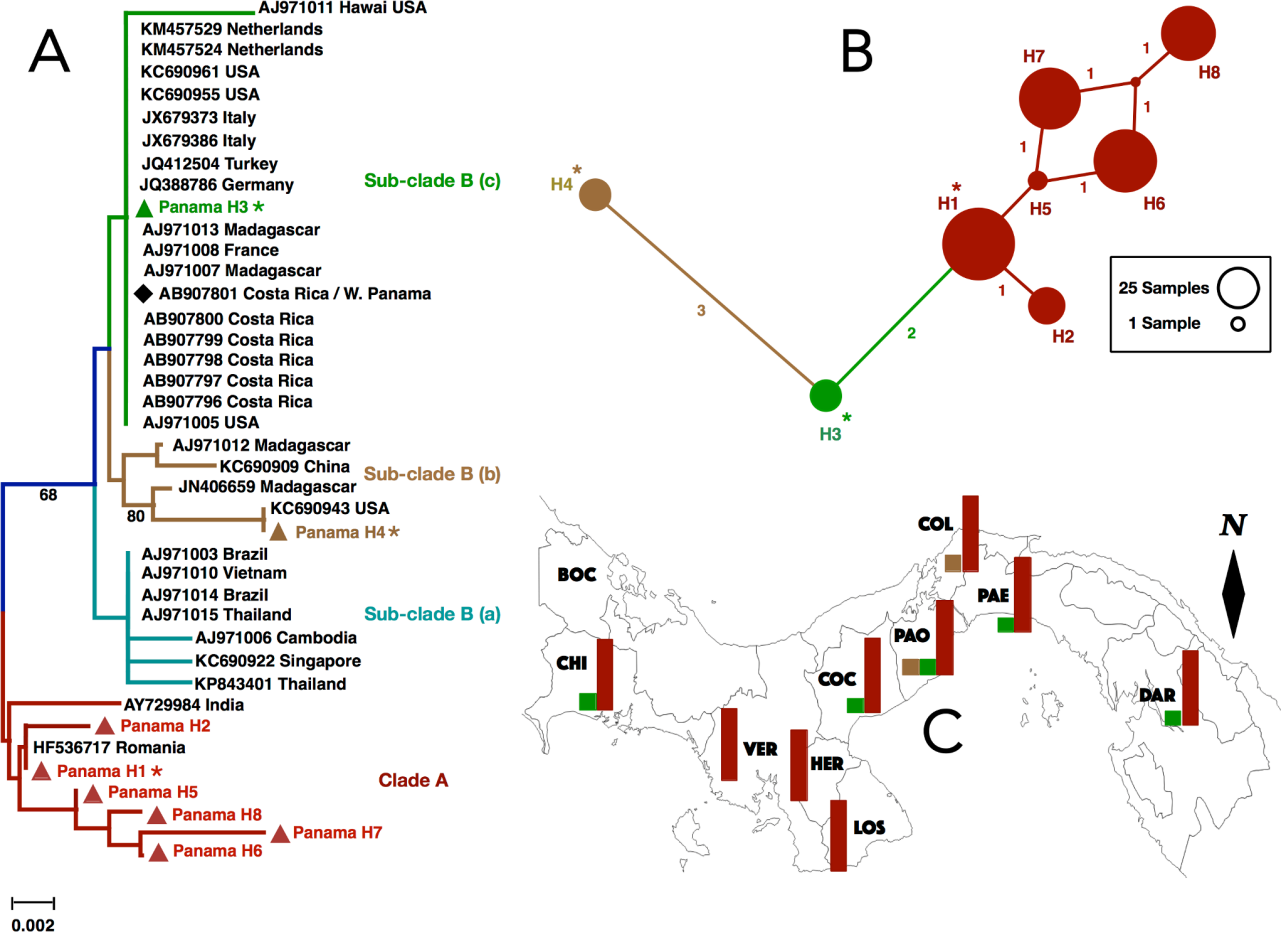
**(A) Neighbor-joining phylogenetic tree of Panamanian and worldwide *CO1* haplotypes of *Aedes albopictus* from GenBank** (http://blast.ncbi.nlm.nih.gov/). Panamanian haplotypes belonging to Clade A, sub-Clade B (b) and sub-Clade B (c) are shown in red, brown and green triangles, respectively. Bootstrap values depicting branch support higher than 60% are shown in the tree. Asterisks (*) in Haplotypes 1, 4 and 3 indicate most frequent Panamanian haplotypes in Clade A, sub-Clade B (b) and sub-Clade B (c), respectively. Black diamond symbolizes sequence AB907801 that was found in Costa Rica and Western Panama. **(B) TCS network depicting mutational relationships among three *CO1* haplogroups of *Aedes albopictus*.** Haplogroups 1, 2 and 3 mimic the color of Clade A, sub-Clade B (c) and sub-Clade B (b), in that order. Haplotypes are represented by circles and their sizes reflect their population frequencies. One missing haplotype is represented by a red dot in haplogroup 1 and numbers along lines are mutational differences. Haplogroups 1, 2 and 3 match those in Table 3. **(C) Geographic distribution of haplogroups 1 (red), 2 (green) and 3 (brown) across Panama.** Bars correspond to the regional frequency of that haplogroup per sampling Province (see Table 1 for additional details). Black diamond (i.e., North arrow) indicates the direction to the geographic North Pole.

High values of genetic diversity for both mosquito species were obtained from central Panama, in the provinces of Panama (PAO, PAE) and Colon (COL) (Table 2). *Aedes albopictus* also had high genetic diversity in Chiriquí (CHI), in western Panama near the Costa Rican border. In contrast, the lowest values of genetic diversity in populations of these mosquitoes were detected from the Azuero Peninsula, in Coclé (COC) and Herrera (HER) for *Ae*. *aegypti*, and in HER and Los Santos (LOS) for *Ae*. *albopictus* (Table 2; Figs 2 and 3). Values of Tajima’s *D* test were positive and not significantly different from zero in either species (S1 Table). This is consistent with long-term stable population size in both species (i.e., demographic equilibrium). The mismatch frequency distributions did not fit a model of population expansion in either species, again coherent with the expectation of long-term stable effective populations (S1 Table; S2 Fig).

### The number of founder maternal lineages of *Aedes* mosquitoes into Panama

Haplotype networks inferred for *Ae*. *aegypti* using different methods depicted similar topologies and relationships. These networks displayed two core haplotypes (H1 and H13) separated by roughly 10 mutational steps and surrounded by low and intermediate frequency variants (Fig 2B; S3 Fig). These two deeply divergent haplogroups that occupied peripheral positions in the networks, were the most frequent (e.g., H1 = 43.4%, H13 = 21.3%) and widespread, being detected in 10 provinces (Table 3; Fig 2C). In addition, three low frequency and closely related haplotypes (e.g., H7, H8 and H9), representing a third haplogroup, were separated from H1 and H13 by 9 to 12 mutational steps, respectively (Fig 2B). This last haplogroup was missing from 60% of the sampling area and completely absent from western Panama and from the Azuero Peninsula (Table 3; Fig 2C). The average number of pairwise differences between these three variants (e.g., Haplogroup 1 = H1-H6; Haplogroup 2 = H10-H13; Haplogroup 3 = H7-H9) were *k* = 4.667, *k* = 2.192 and *k* = 1.234, respectively. Nucleotide divergence ranged from 0.014 between haplogroups 1 and 3 to 0.018 between haplogroups 1 and 2. Values of Tajima’s *D* were negative for haplogroup 1 and positive for haplogroup 2, but neither was statistically significantly different from zero (S1 Table).

**Table 3:** Heat map depicting the geographic distribution and frequency of *CO1* haplotypes of three haplogroups of *Ae*. *aegypti* and three haplogroups of *Ae*. *albopictus* found in Panama.

Locality	Province	*Aedes aegypti*													*Aedes albopictus*							
		1						2				3			1						2	3
		H1	H2	H3	H4	H5	H6	H10	H11	H12	H13	H7	H8	H9	H1	H2	H5	H6	H7	H8	H3	H4
1. Guabito	BOC	**4**																				
2. Isla Colon		**2**																				
3. Nuevo Almirante		**1**									**3**											
4. Aserrio	CHI	**3**						**1**			**3**				**3**					**2**		
5. El progreso																	**2**		**1**		**2**	
6. San José		**6**									**2**				**2**			**2**				
7. San José	VER	**1**			**2**						**3**				**2**			**1**		**1**		
8. San Pablo,															**3**					**1**		
9. San Francisco		**2**	**2**								**2**				**1**				**2**	**2**		
10. Parita	HER	**4**								**2**									**6**			
11. San Juan Bautista		**5**																**2**	**4**			
12. La Villa	LOS	**1**									**2**							**1**	**2**			
13. Macaracas										**2**	**3**							**6**				
14. Las Tablas				**3**														**3**				
15. Barrios unidos	COC	**3**								**1**												
16. Aguadulce		**4**																**2**		**4**	**1**	
17. Antón		**2**						**2**										**2**	**2**	**2**		
18. Burunga	PAO	**1**	**2**		**1**										**2**			**1**			**1**	
19. Los Cerezos 2											**1**			**3**				**2**				**1**
20. Nuevo Emperador		**2**	**1**				**1**		**1**						**2**					**3**		
21. Las Garzas	PAE	**2**									**1**		**1**		**3**					**1**		
22. Chorrillo					**1**						**1**				**2**					**1**		
23. Camino Omar							**1**						**1**		**1**	**2**		**1**				
24. San Pablo		**2**	**1**												**1**	**1**					**1**	
25. El Valle		**2**		**1**		**1**														**2**		**1**
26. Buena Vista	COL	**2**									**1**				**2**							**1**
27. Puerto pilón						**1**			**2**			**1**			**2**	**1**			**1**			
28. Villa del Caribe				**1**			**2**				**1**	**2**			**2**	**1**		**1**				**1**
29. Bello horizonte	DAR	**2**									**2**		**2**		**3**	**3**					**1**	
30. Piedra candela						**1**					**1**				**1**					**4**		

Haplogroup 1 (Haplo 1 = H1, H2, H3, H4, H5, H6), haplogroup 2 (Haplo 2 = H10, H11, H12 and H13) and haplogroup 3 (Haplo 3 = H7, H8, H9) of *Ae*. *aegypti* as well as haplogroup 1 (Haplo 1 = H1, H2, H5, H6, H7, H8), haplogroup 2 (Haplo 2 = H3) and haplogroup 3 (Haplo 3 = H4) of *Ae*. *albopictus* are shown in black and grey color, respectively.

Haplotype networks inferred for *Ae*. *albopictus* using different methods were congruent in topology and the relationships between variants, representing three main haplogroups. Haplogroup 1 depicted four high (e.g., H1, H6, H7, H8) and two low frequency (e.g., H2 and H5), but closely related variants, being separated from each other by one mutational step. This haplogroup was the most frequent (e.g., H1 = 27.3% and H6 = 20.5%) and widespread, being present in 10 provinces (Table 3; Fig 3C). In addition, two low frequency and genetically divergent haplotypes (e.g., H3 and H4) (Fig 3B; S4 Fig) corresponded to haplogroups 2 and 3, in that order. These haplogroups were separated from each other by two missing mutations and from haplogroup 1 by three mutational steps; they were infrequent and absent from most of the Azuero Peninsula (VER, HER and LOS) (Table 3; Fig 3C). The average number of pairwise differences between designated haplogroups (e.g., Haplogroup 1 = H1, H2, H5, H6, H7, H8; Haplogroup 2 = H3, and Haplogroup 3 = H4) were *k* = 1.853, *k* = 0, and *k* = 0, respectively. Nucleotide divergence ranges from 0.0065 between haplogroups 2 and 3 to 0.0117 between haplogroups 1 and 3. Values of Tajima’s *D* were all positive and not statistically significantly different from zero (S1 Table).

### Geographic origin and distribution of invading *Aedes* mosquitoes in Panama

A NJ phylogenetic tree from the combined dataset of Panamanian and GenBank haplotypes of *Ae*. *aegypti* (S2 Table; S3 Fig) depict two well supported mitochondrial clades (i.e., maternal lineages A and B). Panamanian sequences in these clades were associated with others from tropical areas around the world. Clade A, which included the most frequent Panamanian haplotype (H1), was further subdivided into two divergent and well bootstrap-supported Sub-clades (Fig 2A; S3 Fig). Sub-clade A (a) included three haplotypes (e.g., H7, H8, H9) that matched others from East Africa (e.g., Tanzania), South America (e.g., Bolivia, Colombia), Europe Island (e.g., near Madagascar in the Indian Ocean), Asia (e.g., Vietnam) and the Caribbean region (e.g., Martinique) (Fig 2A). Sub-clade A (b) included 6 haplotypes (e.g., H1, H2, H3, H4, H5, H6) that grouped together with others from North America (e.g., USA, Mexico), South America (e.g., Brazil), Asia (e.g., Thailand) and the Caribbean region (e.g., Martinique). In addition, Clade B, which included the second most frequent Panamanian haplotype (H13), comprised four Panamanian haplotypes (e.g., H10, H11, H12, H13) that grouped together with others from Europe (e.g., Portugal), West Africa (e.g., Cameroon, Guinea, Rep. Ivory Coast), South America (e.g., Colombia, Venezuela), North America (e.g., USA, Mexico), Asia (e.g., Cambodia, India, Thailand, Vietnam) and the French Polynesia (e.g., near the island of Tahiti in the South Pacific Ocean). The most common and genetically distinct Panamanian haplotypes of *Ae*. *aegypti* were located in the NJ tree near sequences from Martinique (H1 –in Sub-clade A—b), Vietnam (H8—in Sub-clade A—a), and Thailand (H13 –in Clade B), respectively (Fig 2A; S3 Fig). Sub-clade A (b) and Clade B were sympatric and widely distributed across the Isthmus of Panama, but the former seems more predominant. In contrast, Sub-clade A (a) was missing from most of western Panama, including the Azuero Peninsula (Fig 2C).

The NJ phylogenetic tree from the combined dataset of Panamanian and GenBank haplotypes of *Ae*. *albopictus* (S3 Table; S4 Fig) depicted two well supported mitochondrial clades (e.g., maternal lineages A and B). Panamanian sequences in these two clades were both associated with others from tropical and temperate regions (Fig 3A; S4 Fig). Clade A comprised 6 haplotypes (e.g., H1, H2, H5, H6, H7 and H8) that grouped together with others from tropical India and temperate Romania including the most frequent Panamanian haplotype (H1). Clade B was further subdivided into three smaller, and moderately supported, but somewhat divergent Sub-clades (Fig 3A; S4 Fig). Sub-clades B (b) and B (c) each included a single Panamanian haplotype (e.g., H4 and H3, respectively) that either clustered with sequences from the USA and Madagascar, or with a wide range of other countries including neighboring Costa Rica, respectively. Both, Sub-clade B (b) and Sub-clade B (c), encompassed haplotypes from temperate and tropical regions, but the former was more restricted to tropical Madagascar, whereas the latter encompassed mainly haplotypes from temperate European countries. A third Sub-clade within Clade B (i.e., Sub-clade B—a) included sequences from Asia (e.g., Thailand, Vietnam, Cambodia, Singapore) and from Brazil, but did not comprise Panamanian haplotypes. The most common and genetically distinct Panamanian haplotypes of *Ae*. *albopictus* were located in the NJ tree near sequences from Romania (H1 –in Clade A), Germany (H3 –in Sub-clade B—c), and the USA (H4 –in Sub-clade B—b), respectively. Additional phylogenetic analysis using mitogenome *CO1* sequences of *Ae*. *albopictus* [36] reinforced our findings (S1 Text). Clade A was prevalent and widely distributed across the Panamanian Isthmus while Sub-clade B (b) and Sub-clade B (c) were found in low frequency and were absent from more than 50% of the sampling area, including the Azuero Peninsula. Panamanian representatives of these mitochondrial clades were absent from the province of Bocas del Toro (BOC).

## Discussion

Phylogeographic data from Panamanian populations of *Ae*. *aegypti* and *Ae*. *albopictus* elucidate key components of their demographic history. We found evidence for several maternal lineages in these two mosquito vectors, perhaps reflecting multiple colonization events of the Isthmus of Panama, and ancestral polymorphisms within their native Old World range, or both. The signal of mutation-drift equilibrium in Panamanian populations of *Ae*. *aegypti* and *Ae*. *albopictus* could likely be an artifact of pooling historically diverged, yet recently admixed invasive populations together [27].

### Invasion history of *Aedes* (Stegomyia) *aegypti* (Linnaeus 1762) and *Aedes* (Stegomyia) *albopictus* (Skuse 1894) into Panama

We detected members of two major mitochondrial clades in samples of *Ae*. *aegypti* from Panama, one of which (i.e., Clade A) was further subdivided into two Sub-clades. Each of these three molecular clusters includes a separate set of Panamanian haplotypes, supporting the pattern of high genetic diversity observed for this invasive species around the world [19,25,46]. Our results are consistent with prior studies that suggested that *Ae*. *aegypti* invaded the Americas during multiple introduction events [24]. Two deeply divergent mitochondrial clades originating from West and East Africa, respectively, likely corresponding to mtDNA clades A and B in this study, are thought to be the invaders in Europe, Asia and the Americas [19]. Haplotypes in clade B were thought to be genetically similar to mosquitoes from West Africa, and based on the prevalence of historical sea trade from this region, assumed to be the first American invaders. Moreover, members of clade B were found in low frequency in Brazil and hypothesized to have survived OPS eradication programs in the 1950s [19,22,24,25]. Nevertheless, more recent studies suggested that deep-divergence in the mitochondrial genome of *Ae*. *aegypti* is most likely due to ancient population processes within its native range in Africa, rather than due to signatures of its global population expansion [26]. Bennett and others [27] demonstrates that these mitochondrial clades co-exist across continental Africa, and thus, they likely represent signals of retained ancestral mitochondrial polymorphisms among recently admixed populations. In view of this finding, the African origins of Panamanian members of clades A and B cannot be established with certainty here, nor can we claim that their co-existence in Panama is the result of two separate introduction events.

In addition, our results indicate the existence of geographic structure within Clade A. Panamanian members of Sub-clade A (b) of *Ae*. *aegypti* are common and widely-distributed across the country, suggesting that these populations have persisted locally for longer. In contrast, members of Sub-clade A (a) are missing from the Azuero Peninsula possibly due to a more recent invasion time, or due to environmental or biological barriers hampering successful colonization of this area (Fig 1). Therefore, we posit that *Ae*. *aegypti* invaded the Isthmus of Panama at least twice, perhaps at different periods and also possibly via different introduction routes. Future studies will need to use high-resolution multi-locus genomic data along with a wider sampling scheme that includes historical samples of *Ae*. *aegypti* in order investigate whether members of Sub-clade A (a) were the first invaders in Panama, and to determine whether or not Sub-clades A (a) and A (b) survived the OPS eradication program.

As in *Ae*. *aegypti*, we recovered two major mitochondrial clades among samples of *Ae*. *albopictus* from Panama, one of which (i.e., Clade B) was further subdivided into three sub-clades. Three of these four molecular clusters included a discrete set of Panamanian haplotypes supporting a pattern of high genetic diversity that has been noticed for this invasive species within the Americas [3,11]. In agreement with previous studies, these molecular clusters consisted of representatives from both temperate and tropical regions. A previous study hypothesized that Panamanian population of *Ae*. *albopictus* originated entirely from tropical Costa Rica [37]. Here, we posit that colonization of *Ae*. *albopictus* into Panama is ongoing, with a minimum of three maternal lineages having invaded the country at different times and through different introduction routes in just over 10 years. Panamanian sequences in Clade A were widely distributed and prevalent across the Isthmus of Panama, consistent with a single origin of Clade A as a result of the first introduction event in 2004. In contrast, Panamanian samples representing Sub-clades B (b) and B (c) were uncommon throughout the country and entirely absent from the Azuero Peninsula. Therefore, they appear to have arrived more recently in Panama. Clade A and Sub-clade B (b) were likely introduced through cargo vessels from Europe and from the United States, respectively. Sub-clade B (c) was probably introduced through the shipment of used tires via land transportation from Costa Rica, as anticipated in previous studies [28,37]. However, to corroborate theses hypotheses, future studies will need to use high-resolution multi-locus genomic data and wider sampling scheme, including specimens from *Ae*. *albopictus* native’s range in Asia, to rule out a possible confounding role of shared ancestral mitochondrial polymorphisms in this species as well.

### Comparison of the demographic histories of *Aedes* mosquitoes

Although the population genetic history of *Ae*. *aegypti* and *Ae*. *albopictus* were similar in that both species consists of multiple maternal lineages in Panama, there are noteworthy differences in their demographic histories. Estimated nucleotide diversity was higher in *Ae*. *aegypti* likely due to differences in historical demography, which could include an earlier introduction into Panama or a large invading pool. However, as pointed out previously [27], genetic diversity in this vector is affected by shared ancestral polymorphisms from the native range of invasive populations. Ever since its first invasion more than a century ago, *Ae*. *aegypti* may have accumulated high levels of molecular variation through adaptation to diverse environmental conditions and selective pressures such as insecticide control or interspecific competition with *Ae*. *albopictus*. Importantly, our results strongly suggest that at least one introduction of *Ae*. *aegypti* occurred into the Isthmus of Panama subsequent to the eradication efforts in 1950, which could have further contributed to an increased level of nucleotide diversity in this species. However, we cannot rule out the possibility that this species also managed to persist in some local areas through the period of eradication. In contrast, *Ae*. *albopictus* is a more recent invader in Panama; since it’s introduction in 2004, this species is still in the early stages of the colonization process and is likely adapting to environmental conditions and selective pressures across the country [28]. Curiously, estimates of haplotype diversity did not differ statistically between these two mosquitoes, despite their different introduction times. Even more surprisingly, the number of mitochondrial haplotypes of *Ae*. *albopictus* that we recovered in Panama is higher than that reported previously in recently invaded countries from Africa, Europe and the Americas [9,29,31,47]. We posit here that this observation could be due to a much faster invasion mode by *Ae*. *albopictus*, which could have experienced multiple introduction events within a very short period of time (i.e., 12 years). If each of these events involved mosquitoes from different geographic regions of the world (i.e., temperate and tropical), it would explain the relatively elevated level of haplotype diversity we observed in *Ae*. *albopictus* from Panama. *Aedes aegypti* also invaded Panama in at least two occasions, but these introductions likely involved founder populations from neighboring tropical environments across a longer period of time. Finally, our results reinforce the notion that *Ae*. *albopictus* has a broader ecological tolerance as compared to *Ae*. *aegypti*, and provides a demonstrable, local consequence (high local genetic diversity) of such ecological adaptability. *Aedes albopictus* colonized 80% of the world’s available habitat during the last three decades, and its high-speed rate of expansion and colonization has allowed it to reach places where *Ae*. *aegypti* does not currently exist [8].

The highest genetic diversity for both species was gathered from Panama Este (PAE) and Panama Oeste (PAO) and Colon (COL) provinces, which are the Pacific and Atlantic entries of the Panama Canal, respectively. Also, *Ae*. *albopictus* depicted moderately high genetic diversity in Chiriqui near the Costa Rican border. These shared outcomes suggest that both *Aedes* invaded Panama assisted by humans, likely through the trade of commercial goods (i.e., used tires). This trend was perhaps facilitated by the numerous water and land inter-connections across most of the country. Most *Aedes* introductions into Panama are likely to have occurred via marine cargo through the Panama Canal, but at least one introduction of *Ae*. *albopictus* has probably occurred via land transportation from Costa Rica [28,37]. Furthermore, some haplogroups in both species were missing from the Azuero Peninsula. This could be due to potential climatic or biological barriers experienced by these mosquitoes there, where seasonally dry conditions are recorded every year (Fig 1). This is unlikely to be the result of competition between species though, given that *Ae*. *albopictus* only invaded this region in 2015. Since this is likely a recent invasion event, there had not been enough time for rare (i.e., low frequency) haplotypes in Panama to colonize the Azuero Peninsula.

Our single-marker comparative population genetic approach has some limitations for inferring full population history of *Aedes* mosquitoes in Panama. These include the potential presence of *numts* (i.e., nuclear copies of the mitochondrial genome) in the dataset, and the different sizes of nucleotide fragment of the *CO1* gene analyzed between species. We believe that these issues are not meaningfully influencing our results. First, the *CO1* gene has been used thoroughly in the past to investigate the invasion histories of *Aedes* mosquitoes around the world, with very high success. Moreover, as in the majority of past studies about this subject, we did not detect an elevated rate of non-synonymous mutations, stop codons or multiple peaks in our chromatograms or sequences, which would indicate the presence of *numts*. Second, the range of values of nucleotide and haplotype diversity for Panamanian populations of both species are similar to those reported before by a wide range of studies comprising different mitochondrial genes, nuclear coding regions, microsatellites, primers, and countries around the world [9,22–25,29,30]. Finally, we did not test for the presence of the bacterium *Wolbachia* in *Aedes* specimens from Panama, and thus the possibility that our outcomes are influenced by this factor to some degree cannot be ruled out without further testing especially in *Ae*. *albopictus*, which is regularly found infected with *Wolbachia*.

### Implications for vector control and for the transmission of emergent arboviruses in Panama

The mitochondrial haplogroups of *Ae*. *aegypti* and *Ae*. *albopictus* in this study have distinctive genetic backgrounds and possibly different geographic origins. Therefore, they could display biological, ecological, and behavioral traits that might potentially result in different vector competence and/or insecticide resistance profiles. Consequently, trying to understand the molecular differences within and between *Ae*. *aegypti* and *Ae*. *albopictus*, plus the biological mechanism by which genetic diversity arrives to and get shuffled around the Isthmus of Panama, might aid to deciphering the species’ role in arbovirus transmission as well as the prospect of vector control programs targeting these mosquitoes.

A trial release of the Oxitec transgenic mosquito line (OX513A) exclusively targeting *Ae*. *aegypti* was recently undertaken in communities of western Panama City. The study by Gorman and others [48] reported a reduction of roughly 80% of the native *Ae*. *aegypti* population in approximately four months of continuous release. Despite the success of the transgenic approach in Panama, it took almost six months and more than five million male mosquitoes for the transgenic line to bring natural populations of *Ae*. *aegypti* down to eradication levels in two small areas. Furthermore, an important population parameter to assess the long-term sustainability of this approach, namely the immigration rate from neighboring untreated areas has not been reported for *Ae*. *aegypti* prior to our study. Our findings corroborate the ability of *Aedes* mosquitoes to reinvade Panama likely assisted by sea and land transportation of human goods. Moreover, novel genetic diversity introduced into the country can quickly spread across space through the road network system [28], which suggests that in Panama, eradication plans relying on transgenic mosquitoes will require constant and permanent inputs of transgenic males to maintain *Ae*. *aegypti* populations at eradication levels, even at local scales.

In addition, populations of *Ae*. *albopictus* in Panama could have a similar genetic makeup to temperate Europe, which may favor transmission of certain CHIKV genotypes over others, with consequences for the emergence of epidemics [49]. Also, given that transovarial transmission of CHIKV is possible by *Ae*. *albopictus* [50], infected European progenies could get introduced and quickly spread across Panama triggering serious CHIKV epidemics. Therefore, future studies on the population genomics of these mosquitoes will need to estimate gene flow and immigration rates at transcontinental and local geographic scales to inform vector suppression strategies and to clarify the role of *Aedes* species in arbovirus transmission.

The within species molecular diversity observed in *Aedes* mosquitoes from Panama could underlie their ability to compete for habitat and to interact at the intra-specific level, which could also interfere with vector control strategies such as vector suppression. Our data presents a great opportunity for Panamanian scientists to address scientific questions about inter and intra-species ecological and epidemiological differences in these two mosquito vectors. A scientific study addressing the trade of used tires into and across the country should further assist in deciphering the role of human-assisted dispersal as a major factor shaping the distribution of genetic diversity of *Aedes* mosquitoes across the Isthmus of Panama. For now, there is a need to target spare tires for eradication to avoid the introduction of novel mutations that might enhance the transmission of CHIKV and ZIKV in Panama.

## Supporting information

S1 Table Metrics of population genetic diversity for *Aedes* mosquitoes in Panama based on partial *CO1* gene sequences. *Hd* = haplotype diversity; *π* = nucleotide diversity. Values of Tajima’s D test are shown for each Clade and haplogroups. N/A means that no result was obtained when estimating population metrics due to low number of sequences in those groupings.
(XLSX)

S2 Table Geographic origin (i.e., country) and GenBank accession codes of *CO1* sequences of *Aedes aegypti* used herein in phylogeographic analysis. Sequences from GenBank include haplotypes of *Ae*. *aegypti* from Africa, Asia, North-Central and South America as well as from the Caribbean region.
(DOC)

S3 Table Geographic origin (i.e., country) and GenBank accession codes of *CO1* sequences of *Aedes albopictus* used herein in phylogeographic analysis. Sequences from GenBank include haplotypes of *Ae*. *albopictus* from Asia, America, Europa and the Pacific Ocean.
(DOCX)

S1 Text Data and methods used in complementary Phylogenetic reconstruction with partial *CO1* gene sequences of *Aedes albopictus* from Battaglia *et al*. [36]. Sequences from GenBank that did not meet the selection criteria are also included here.
(DOCX)

S1 Fig Map of sampling localities in Panama. Circles represent the locations of each locality. Black and red circles represent the presence of both or only one species. The numbers represent the locality in Table 1.
(TIFF)

S2 Fig **Mismatch distribution of *CO1* sequences of *Aedes* mosquitoes of Panama (A: *Ae*. *aegypti* and B: *Ae*. *albopictus*).** 122 sequences comprising 728 base pairs for *Aedes aegypti* and 117 sequences comprising 461 bp from *Aedes albopictus* were used in these analyses.
(TIFF)

S3 Fig **(A) Neighbor-joining phylogenetic tree of Panamanian and worldwide *CO1* haplotypes of *Aedes aegypti* from GenBank** (http://blast.ncbi.nlm.nih.gov/). Panamanian haplotypes belonging to Clade A and Clade B are shown in red and blue triangles, respectively. Bootstrap values depicting branch support higher than 60% are shown in the tree. Asterisk in Haplotype 1 and Haplotype 13 indicate most frequent Panamanian variants within Clade A and B, in that order. (B–F / left to right) Haplotype relationships of *Ae*. *aegypti* given by The Minimum Spanning (MS), Median Joining (MJ), Tight Span Walker (TSW), Integer NJ Net (INJN) and Parsimony (TCS) networks, respectively.
(PPTX)

S4 Fig **(A) Neighbor-joining phylogenetic tree of Panamanian and worldwide *CO1* haplotypes of *Aedes albopictus* from GenBank** (http://blast.ncbi.nlm.nih.gov/). Panamanian haplotypes belonging to Clade A and Clade B are shown in red and blue triangles, respectively. Bootstrap values depicting branch support higher than 60% are shown in the tree. Asterisks (*) in Haplotype 1 and Haplotype 4 indicate most frequent variants in Clade A and B, respectively. (B–F / left to right) Haplotype relationships of *Ae*. *albopictus* given by The Minimum Spanning (MS), Median Joining (MJ), Tight Span Walker (TSW), Integer NJ Net (INJN) and Parsimony (TCS) networks, respectively.
(PPTX)
