# Supplementary material for: Maternal invasion history of Aedes aegypti and Aedes albopictus into the Isthmus of Panama: Implications for the control of emergent viral disease agents
Source: PLoS One. 2018 Mar 26;13(3):e0194874. doi: 10.1371/journal.pone.0194874 (PMC5868824; doi:10.1371/journal.pone.0194874)
Supplement: S2 Table — Sequences from GenBank include haplotypes of Ae. aegypti from Africa, Asia, North-Central and South America as well as from the Caribbean region. (DOC) [file pone.0194874.s002.doc]

| **Geographical Area** | **Country** | **Climate** | **Accession Number** | **References** |
| --- | --- | --- | --- | --- |
| Central Africa | Cameroon | Tropical | JQ926702 | Paupy C., et al (2012) |
| West Africa | Rep. Ivory Coast | Tropical | JQ926695 | Paupy C., et al (2012) |
|  | Rep. Ivory Coast | Tropical | JQ926693 | Paupy C., et al (2012) |
|  | Guinea | Tropical | JQ926700 | Paupy C., et al (2012) |
| Eastern Africa | Tanzania | Tropical | JQ926704 | Paupy C., et al (2012) |
| Southeast Asia | Thailand | Tropical | JQ926692 | Paupy C., et al (2012) |
|  | Thailand | Tropical | JQ926691 | Paupy C., et al (2012) |
|  | Vietnam | Tropical | JQ926686 | Paupy C., et al (2012) |
|  | Vietnam | Tropical | HQ688292 | Fort P., et al (2012) |
|  | Vietnam | Tropical | JQ926685 | Paupy C., et al (2012) |
|  | Cambodia | Tropical | HQ688294 | Fort P., et al (2012) |
|  | Cambodia | Tropical | JQ926688 | Paupy C., et al (2012) |
|  | Cambodia | Tropical | JQ926689 | Paupy C., et al (2012) |
| South Asia | India | Tropical | KT339683 | Vadivalagan C., et al (2016) |
| Caribbean | Martinique | Tropical | JQ926696 | Paupy C., et al (2012) |
|  | Martinique | Tropical | JQ926697 | Paupy C., et al (2012) |
| South America | Venezuela | Tropical | JQ926701 | Paupy C., et al (2012) |
|  | Brazil | Tropical | JQ926703 | Paupy C., et al (2012) |
|  | Colombia | Tropical | KM203201 | Jaimes-Duenez J. et al. (2015) |
|  | Colombia | Tropical | KM203151 | Jaimes-Duenez J. et al. (2015) |
|  | Colombia | Tropical | KM203188 | Jaimes-Duenez J. et al. (2015) |
|  | Colombia | Tropical | KM203141 | Jaimes-Duenez J. et al. (2015) |
|  | Bolivia | Tropical | JQ926676 | Paupy C., et al (2012) |
| North America | Mexico | Tropical | JQ926698 | Paupy C., et al (2012) |
|  | Mexico | Tropical | JQ926699 | Paupy C., et al (2012) |
|  | USA | Temperate | EU352212 | ***** Lobo N., et al (2007) |
|  | USA | Temperate | JQ926684 | Paupy C., et al (2012) |
| Indian Ocean | Europa Island | Temperate | HQ688296 | Fort P., et al (2012) |
| South Pacific Ocean | French Polynesia | Temperate | HQ688295 | Fort P., et al (2012) |
| Southwestern Europe | Portugal | Temperate | KF909122 | Seixas G., et al (2013) |

*References in* S2 Table

**Paupy C, Le Goff G, Brengues C, Guerra M, Revollo J, Barja Simon Z, et al.** Genetic structure and phylogeography of Aedes aegypti, the dengue and yellow-fever mosquito vector in Bolivia. Infect Genet Evol. 2012;12: 1260–1269. doi:10.1016/j.meegid.2012.04.012 PMID: 22522103

**Fort P, Albertini A, Van-Hua A, Berthomieu A, Roche S, Delsuc F, et al.** Fossil rhabdoviral sequences integrated into arthropod genomes: ontogeny, evolution, and potential functionality. Mol Biol Evol. 2012 Jan; 29(1):381–90. doi: 10.1093/molbev/msr226. PMID: 21917725

**Vadivalagan C, Karthika P, Murugan K, Panneerselvam C, Paulpandi M, Madhiyazhagan P, et al.** Genetic deviation in geographically close populations of the dengue vector Aedes aegypti (Diptera: Culicidae): influence of environmental barriers in South India. Parasitol Res. 2016 Mar;115(3):1149–60. doi: 10.1007/s00436-015-4847-7. PMID: 26627691

**Jaimes-Dueñez J, Arboleda S, Triana-Chávez O, Gómez-Palacio A.** Spatio-temporal distribution of Aedes aegypti (Diptera: Culicidae) mitochondrial lineages in cities with distinct dengue incidence rates suggests complex population dynamics of the dengue vector in Colombia. PLoS Negl Trop Dis. 2015;9: e0003553. doi:10.1371/journal.pntd.0003553 PMID: 25893246

**Seixas G, Salgueiro P, Silva AC, Campos M, Spenassatto C, Reyes-Lugo M, et al.** Aedes aegypti on Madeira Island (Portugal): genetic variation of a recently introduced dengue vector. Mem Inst Oswaldo Cruz. Fundação Oswaldo Cruz; 2013;108 Suppl 1(suppl 1):3–10. doi: 10.1590/0074-0276130386. PMID: 24473797.

**Lobo, N.F., Lovin, D., DeBruyn, B., Puiu, D., Shumway, M., Haas, B., Nene, V. and Severson, D.W.** The mitochondrial genome of the Yellow fever mosquito – Aedes aegypti. Unpublished. 2007, GenBank direct submission *****.
