# Supplementary material for: Maternal invasion history of Aedes aegypti and Aedes albopictus into the Isthmus of Panama: Implications for the control of emergent viral disease agents
Source: PLoS One. 2018 Mar 26;13(3):e0194874. doi: 10.1371/journal.pone.0194874 (PMC5868824; doi:10.1371/journal.pone.0194874)
Supplement: S3 Table — Sequences from GenBank include haplotypes of Ae. albopictus from Asia, America, Europa and the Pacific Ocean. (DOCX) [file pone.0194874.s003.docx]

| **Geographical Area** | **Country** | **Climate** | **Accession Number Number** | **References** |
| --- | --- | --- | --- | --- |
| Southwest Indian Ocean | Madagascar | Sub-Tropical | AJ971007 | Mousson L., et al (2005) |
|  | Madagascar | Sub-Tropical | JN406659 | Raharimalala FN., et al (2012) |
|  | Madagascar | Sub-Tropical | AJ971012 | Mousson L., et al (2005) |
|  | Madagascar | Sub-Tropical | AJ971013 | Mousson L., et al (2005) |
| South Asia | India | Tropical | AY729984 | Kumar NP., et al (2007) |
| Southeast Asia | Cambodia | Tropical | AJ971006 | Mousson L., et al (2005) |
|  | Thaïland | Tropical | AJ971015 | Mousson L., et al (2005) |
|  | Thailand | Tropical | KP843401 | * Sumruayphol,S., et al (2015) |
|  | Vietnam | Tropical | AJ971010 | Mousson L., et al (2005) |
|  | China | Tropical | KC690909 | Zhong D., et al (2013) |
|  | Singapore | Tropical | KC690922 | Zhong D., et al (2013) |
| Western Asia | Turkey | Temperate | JQ412504 | Oter K., et al (2013) |
| Central America | Costa Rica | Tropical | AB907796 | Futami K., et al (2015) |
|  | Costa Rica | Tropical | AB907797 | Futami K., et al (2015) |
|  | Costa Rica | Tropical | AB907800 | Futami K., et al (2015) |
|  | Costa Rica | Tropical | AB907798 | Futami K., et al (2015) |
|  | Costa Rica | Tropical | AB907799 | Futami K., et al (2015) |
|  | Panama | Tropical | AB907801 | Futami K., et al (2015) |
| South America | Brazil | Tropical | AJ971003 | Mousson L., et al (2005) |
|  | Brazil | Tropical | AJ971014 | Mousson L., et al (2005) |
| North America | USA | Temperate | KC690943 | Zhong D., et al (2013) |
|  | USA | Temperate | KC690955 | Zhong D., et al (2013) |
|  | USA | Temperate | KC690961 | Zhong D., et al (2013) |
|  | USA | Temperate | AJ971005 | Mousson L., et al (2005) |
| North Pacific Ocean | USA (Hawaï) | Sub-Tropical | AJ971011 | Mousson L., et al (2005) |
| Central Europe | Germany | Temperate | JQ388786 | ** Kampen H (2012) |
| Southern Europe | Italy | Temperate | JX679373 | Shaikevich E et al (2013) |
|  | Italy | Temperate | JX679386 | Shaikevich E et al (2013) |
| Western Europe | Netherlands | Temperate | KM457524 | Van de Vossenberg et al. (2015) |
|  | Netherlands | Temperate | KM457529 | Van de Vossenberg et al. (2015) |
| West Europe | France | Temperate | AJ971008 | Mousson L., et al. (2005) |
| Southeastern Europe | Romania | Temperate | HF536717 | Prioteasa LF., et al (2015) |

*References in* S3 Table

**Mousson L, Dauga C, Garrigues T, Schaffner F, Vazeille M, Failloux A-B.** Phylogeography of Aedes ( Stegomyia ) aegypti (L.) and Aedes ( Stegomyia ) albopictus (Skuse) (Diptera: Culicidae) based on mitochondrial DNA variations. Genet Res. 2005 Jul 28;86(01):1–11. doi: 10.1017/S0016672305007627 PMID: 16181519.

**Raharimalala FN, Ravaomanarivo LH, Ravelonandro P, Rafarasoa LS, Zouache K, Tran-Van V, et al.** Biogeography of the two major arbovirus mosquito vectors, Aedes aegypti and Aedes albopictus (Diptera, Culicidae), in Madagascar. Parasites & Vectors 2017 10:1. 2012 Mar 20;5:56. doi: 10.1186/1756-3305-5-56 PMID: 22433186.

**Sumruayphol, S., Ruangsittichai, J., Sriwichai, P., Apiwathnasorn,C., Samung, Y. and Dujardin, J.P.** Molecular identification of Aedes scutellaris in Thailand. Unpublished. 2015, GenBank direct submission *****.

**Kumar NP, Rajavel AR, Natarajan R, Jambulingam P.** DNA barcodes can distinguish species of Indian mosquitoes (Diptera: Culicidae). Journal of Medical Entomology. 2007 Jan;44(1):1–7. PMID: 17294914.

**Zhong D, Lo E, Hu R, Metzger ME, Cummings R, Bonizzoni M, et al.** Genetic analysis of invasive Aedes albopictus populations in Los Angeles County, California and its potential public health impact. PLoS ONE. Public Library of Science; 2013;8(7):e68586. doi: 10.1371/journal.pone.0068586. PMID: 23861921.

**Oter K, Gunay F, Tuzer E, Linton Y-M, Bellini R, Alten B.** First record of Stegomyia albopicta in Turkey determined by active ovitrap surveillance and DNA barcoding. Vector Borne Zoonotic Dis. 2013 Oct;13(10):753–61. doi: 10.1089/vbz.2012.1093. PMID: 23808976.

**Futami K, Valderrama A, Baldi M, Minakawa N, Rodríguez RM, Chaves LF.** New and Common Haplotypes Shape Genetic Diversity in Asian Tiger Mosquito Populations from Costa Rica and Panamá. J Econ Entomol. The Oxford University Press; 2015;108: 761–768. doi:10.1093/jee/tou028 PMID: 26470188.

**Kampen H.** Trapping of adult specimens of two invasive mosquito species, Aedes albopictus and Aedes japonicus, and one Mediterranean species, Culiseta longiareolata, in southwestern Germany. Unpublished. 2012, GenBank direct submission **.

**Shaikevich E, Talbalaghi A.** Molecular Characterization of the Asian Tiger Mosquito Aedes albopictus (Skuse) (Diptera: Culicidae) in Northern Italy. International Scholarly Research Notices. Hindawi; 2013 Mar 27;2013(3):1–6. doi: 10.1155/2013/157426.

**Van de Vossenberg BTLH, Ibáñez-Justicia A, Metz-Verschure E, van Veen EJ, Bruil-Dieters ML, Scholte EJ.** Real-time PCR Tests in Dutch Exotic Mosquito Surveys; Implementation of Aedes aegypti and Aedes albopictus Identification Tests, and the Development of Tests for the Identification of Aedes atropalpus and Aedes japonicus japonicus (Diptera: Culicidae). Journal of Medical Entomology. 2015 May;52(3):336–50. doi: 10.1093/jme/tjv020 PMID: 26334807.

**Prioteasa LF, Dinu S, Fălcuţă E, Ceianu CS.** Established Population of the Invasive Mosquito Species Aedes albopictus in Romania, 2012-14. J Am Mosq Control Assoc. 2015 Jun;31(2):177–81. doi: 10.2987/14-6462R PMID: 26181695.
