# Supplementary material for: Maternal invasion history of Aedes aegypti and Aedes albopictus into the Isthmus of Panama: Implications for the control of emergent viral disease agents
Source: PLoS One. 2018 Mar 26;13(3):e0194874. doi: 10.1371/journal.pone.0194874 (PMC5868824; doi:10.1371/journal.pone.0194874)
Supplement: S1 Text — Sequences from GenBank that did not meet the selection criteria are also included here. (DOCX) [file pone.0194874.s004.docx]

**Supplementary text 1 (S1 text)**: This supplementary material includes information about mitochondrial DNA sequences from Vincenza Battaglia et al. (2016), Zhang et al. (2015), Shaikevich et al. (2013) and Raharimalala et al. (2012) that were not included in our Phylogenetic reconstruction of our original submission to Plos One. S1 text also includes mitochondrial DNA sequences that did not meet the selection criteria, and thus they were not used in the latest Phylogenetic analysis requested by reviewers for the revised version of our manuscript to Plos One.

**Additional features of S1 text:** Sequences colored in red are the ones selected for our latest phylogenetic analysis. Sequences colored in blue are the ones from Panama that were included in original phylogenetic analysis as well as in here. The reference position for the *CO1* gene used in S4 Table was the NCBI reference sequence NC_006817.1 that spans from 1674 to 2131 base pairs of a full mitogenome sequence of *Aedes albopictus* (Authors: Ho and Collaborators, 2001). This sequence was also used as the reference sequence in the work of Goubert et al., (2016), which was published in Heredity.

* Correspond to the *CO1* gene sequences from recent publications that were included for the first time in our latest phylogenetic analysis (*See Figure A located after S1 text*). They were extracted from full mitogenome sequences published in Vincenza Battaglia et al. (2016).

† Correspond to the *CO1* gene sequences from Raharimalala F.N. et al. (2012) and Shaikevich E.V. et al. (2013) that were already included in our original NJ phylogenetic tree.

The study conducted by Delatte H. and collaborators (2013) “*Evidence of Habitat Structuring Aedes albopictus Populations in Reunion Island*” did not use *CO1* gene sequences as the molecular marker in their analysis. Instead this study included only analysis with microsatellite sequences. Therefore we did not include data from this study in our latest analysis. We used *CO1* gene sequences from another publication of the same author “*The invaders: Phylogeography of dengue and chikungunya viruses Aedes vectors, on the South West islands of the Indian Ocean*” In Infect Genet Evol (2011).

| **Publication**  **(Author / Year)** | **Genbank**  **Accession numbers** | | **Sequence Length** | **Reference position in the CO1 gene**  **(NC_006817.1)** | **Number of non-overlapping base pairs** | **(%) of overlap with our sequences** |
| --- | --- | --- | --- | --- | --- | --- |
| **Battaglia V., et al. (2016)** | **KX383916** | | **1536** | **1436 - 2972** | **0** | **100** |
| **Battaglia V., et al. (2016)** | **KX383917** | | **1536** | **1436 - 2972** | **0** | **100** |
| **Battaglia V., et al. (2016)** | ***KX383918** | | **1536** | **1436 - 2972** | **0** | **100** |
| **Battaglia V., et al. (2016)** | ***KX383919** | | **1536** | **1436 - 2972** | **0** | **100** |
| **Battaglia V., et al. (2016)** | **KX383920** | | **1536** | **1436 - 2972** | **0** | **100** |
| **Battaglia V., et al. (2016)** | ***KX383921** | | **1536** | **1436 - 2972** | **0** | **100** |
| **Battaglia V., et al. (2016)** | **KX383922** | | **1536** | **1436 - 2972** | **0** | **100** |
| **Battaglia V., et al. (2016)** | ***KX383923** | | **1536** | **1436 - 2972** | **0** | **100** |
| **Battaglia V., et al. (2016)** | ***KX383924** | | **1536** | **1436 - 2972** | **0** | **100** |
| **Battaglia V., et al. (2016)** | **KX383925** | | **1536** | **1436 - 2972** | **0** | **100** |
| **Battaglia V., et al. (2016)** | **KX383926** | | **1536** | **1436 - 2972** | **0** | **100** |
| **Battaglia V., et al. (2016)** | ***KX383927** | | **1536** | **1436 - 2972** | **0** | **100** |
| **Battaglia V., et al. (2016)** | ***KX383928** | | **1536** | **1436 - 2972** | **0** | **100** |
| **Battaglia V., et al. (2016)** | ***KX383929** | | **1536** | **1436 - 2972** | **0** | **100** |
| **Battaglia V., et al. (2016)** | ***KX383930** | | **1536** | **1436 - 2972** | **0** | **100** |
| **Battaglia V., et al. (2016)** | ***KX383931** | | **1536** | **1436 - 2972** | **0** | **100** |
| **Battaglia V., et al. (2016)** | ***KX383932** | | **1536** | **1436 - 2972** | **0** | **100** |
| **Battaglia V., et al. (2016)** | ***KX383934** | | **1536** | **1436 - 2972** | **0** | **100** |
| **Battaglia V., et al. (2016)** | **KX383935** | | **1536** | **1436 - 2972** | **0** | **100** |
| **Battaglia V., et al. (2016)** | **KX809761** | | **1536** | **1436 - 2972** | **0** | **100** |
| **Battaglia V., et al. (2016)** | **KX809762** | | **1536** | **1436 - 2972** | **0** | **100** |
| **Battaglia V., et al. (2016)** | **KX809763** | | **1536** | **1436 - 2972** | **0** | **100** |
| **Battaglia V., et al. (2016)** | ***KX809764** | | **1536** | **1436 - 2972** | **0** | **100** |
| **Battaglia V., et al. (2016)** | ***KX809765** | | **1536** | **1436 - 2972** | **0** | **100** |
| **Zhang et al. (2015)** | ***KR068634** | | **1536** | **1436 - 2972** | **0** | **100** |
| **Shaikevich E.V., et al (2013)** | **†JX679373** | | **687** | **1459 - 2145** | **0** | **100** |
| Shaikevich E.V., et al (2013) | JX679374 | | 687 | 1459 - 2145 | 0 | 100 |
| Shaikevich E.V., et al (2013) | JX679375 | | 687 | 1459 - 2145 | 0 | 100 |
| Shaikevich,E.V., et al (2013) | JX679376 | | 687 | 1459 - 2145 | 0 | 100 |
| Shaikevich,E.V., et al (2013) | JX679377 | | 687 | 1459 - 2145 | 0 | 100 |
| Shaikevich,E.V., et al (2013) | JX679378 | | 687 | 1459 - 2145 | 0 | 100 |
| Shaikevich,E.V., et al (2013) | JX679379 | | 687 | 1459 - 2145 | 0 | 100 |
| Shaikevich,E.V., et al (2013) | JX679380 | | 687 | 1459 - 2145 | 0 | 100 |
| Shaikevich,E.V., et al (2013) | JX679381 | | 687 | 1459 - 2145 | 0 | 100 |
| Shaikevich,E.V., et al (2013) | JX679382 | | 687 | 1459 - 2145 | 0 | 100 |
| Shaikevich,E.V., et al (2013) | JX679383 | | 687 | 1459 - 2145 | 0 | 100 |
| Shaikevich,E.V., et al (2013) | JX679384 | | 687 | 1459 - 2145 | 0 | 100 |
| Shaikevich,E.V., et al (2013) | JX679385 | | 687 | 1459 - 2145 | 0 | 100 |
| **Shaikevich,E.V., et al (2013)** | **†JX679386** | | **687** | **1459 - 2145** | **0** | **100** |
| Beebe N.W., et al (2013) | KC572145 | | 445 | 1634 - 2078 | 56 | 88 |
| Beebe N.W., et al (2013) | KC572146 | | 445 | 1634 - 2078 | 56 | 88 |
| Beebe N.W., et al (2013) | KC572147 | | 445 | 1634 - 2078 | 56 | 88 |
| Beebe N.W., et al (2013) | KC572148 | | 445 | 1634 - 2078 | 56 | 88 |
| Beebe N.W., et al (2013) | KC572149 | | 445 | 1634 - 2078 | 56 | 88 |
| Beebe N.W., et al (2013) | KC572150 | | 445 | 1634 - 2078 | 56 | 88 |
| Beebe N.W., et al (2013) | KC572151 | | 445 | 1634 - 2078 | 56 | 88 |
| Beebe N.W., et al (2013) | KC572152 | | 445 | 1634 - 2078 | 56 | 88 |
| Beebe N.W., et al (2013) | KC572153 | | 445 | 1634 - 2078 | 56 | 88 |
| Beebe N.W., et al (2013) | KC572154 | | 445 | 1634 - 2078 | 56 | 88 |
| Beebe N.W., et al (2013) | KC572155 | | 445 | 1634 - 2078 | 56 | 88 |
| Beebe N.W., et al (2013) | KC572156 | | 445 | 1634 - 2078 | 56 | 88 |
| Beebe N.W., et al (2013) | KC572157 | | 445 | 1634 - 2078 | 56 | 88 |
| Beebe N.W., et al (2013) | KC572158 | | 445 | 1634 - 2078 | 56 | 88 |
| Beebe N.W., et al (2013) | KC572159 | | 445 | 1634 - 2078 | 56 | 88 |
| Beebe N.W., et al (2013) | KC572160 | | 445 | 1634 - 2078 | 56 | 88 |
| Beebe N.W., et al (2013) | KC572161 | | 445 | 1634 - 2078 | 56 | 88 |
| Beebe N.W., et al (2013) | KC572162 | | 445 | 1634 - 2078 | 56 | 88 |
| Beebe N.W., et al (2013) | KC572163 | | 445 | 1634 - 2078 | 56 | 88 |
| Beebe N.W., et al (2013) | KC572164 | | 445 | 1634 - 2078 | 56 | 88 |
| Beebe N.W., et al (2013) | KC572165 | | 445 | 1634 - 2078 | 56 | 88 |
| Beebe N.W., et al (2013) | KC572166 | | 445 | 1634 - 2078 | 56 | 88 |
| Beebe N.W., et al (2013) | KC572167 | | 445 | 1634 - 2078 | 56 | 88 |
| Beebe N.W., et al (2013) | KC572168 | | 445 | 1634 - 2078 | 56 | 88 |
| Beebe N.W., et al (2013) | KC572169 | | 445 | 1634 - 2078 | 56 | 88 |
| Beebe N.W., et al (2013) | KC572170 | | 445 | 1634 - 2078 | 56 | 88 |
| Beebe N.W., et al (2013) | KC572171 | | 445 | 1634 - 2078 | 56 | 88 |
| Beebe N.W., et al (2013) | KC572172 | | 445 | 1634 - 2078 | 56 | 88 |
| Beebe N.W., et al (2013) | KC572173 | | 445 | 1634 - 2078 | 56 | 88 |
| Beebe N.W., et al (2013) | KC572174 | | 445 | 1634 - 2078 | 56 | 88 |
| Beebe N.W., et al (2013) | KC572175 | | 445 | 1634 - 2078 | 56 | 88 |
| Beebe N.W., et al (2013) | KC572176 | | 445 | 1634 - 2078 | 56 | 88 |
| Beebe N.W., et al (2013) | KC572177 | | 445 | 1634 - 2078 | 56 | 88 |
| Beebe N.W., et al (2013) | KC572178 | | 445 | 1634 - 2078 | 56 | 88 |
| Beebe N.W., et al (2013) | KC572179 | | 445 | 1634 - 2078 | 56 | 88 |
| Beebe N.W., et al (2013) | KC572180 | | 445 | 1634 - 2078 | 56 | 88 |
| Beebe N.W., et al (2013) | KC572181 | | 445 | 1634 - 2078 | 56 | 88 |
| Beebe N.W., et al (2013) | KC572182 | | 445 | 1634 - 2078 | 56 | 88 |
| Beebe N.W., et al (2013) | KC572183 | | 445 | 1634 - 2078 | 56 | 88 |
| Beebe N.W., et al (2013) | KC572184 | | 445 | 1634 - 2078 | 56 | 88 |
| Beebe N.W., et al (2013) | KC572185 | | 445 | 1634 - 2078 | 56 | 88 |
| Beebe N.W., et al (2013) | KC572186 | | 445 | 1634 - 2078 | 56 | 88 |
| Beebe N.W., et al (2013) | KC572187 | | 445 | 1634 - 2078 | 56 | 88 |
| Beebe N.W., et al (2013) | KC572188 | | 445 | 1634 - 2078 | 56 | 88 |
| Beebe N.W., et al (2013) | KC572189 | | 445 | 1634 - 2078 | 56 | 88 |
| Beebe N.W., et al (2013) | KC572190 | | 445 | 1634 - 2078 | 56 | 88 |
| Beebe N.W., et al (2013) | KC572191 | | 445 | 1634 - 2078 | 56 | 88 |
| Beebe N.W., et al (2013) | KC572192 | | 445 | 1634 - 2078 | 56 | 88 |
| Beebe N.W., et al (2013) | KC572193 | | 445 | 1634 - 2078 | 56 | 88 |
| Beebe N.W., et al (2013) | KC572194 | | 445 | 1634 - 2078 | 56 | 88 |
| Beebe N.W., et al (2013) | KC572195 | | 445 | 1634 - 2078 | 56 | 88 |
| Beebe N.W., et al (2013) | KC572196 | | 445 | 1634 - 2078 | 56 | 88 |
| Beebe N.W., et al (2013) | KC572197 | | 445 | 1634 - 2078 | 56 | 88 |
| Beebe N.W., et al (2013) | KC572198 | | 445 | 1634 - 2078 | 56 | 88 |
| Beebe N.W., et al (2013) | KC572199 | | 445 | 1634 - 2078 | 56 | 88 |
| Beebe N.W., et al (2013) | KC572200 | | 445 | 1634 - 2078 | 56 | 88 |
| Beebe N.W., et al (2013) | KC572201 | | 445 | 1634 - 2078 | 56 | 88 |
| Beebe N.W., et al (2013) | KC572202 | | 445 | 1634 - 2078 | 56 | 88 |
| Beebe N.W., et al (2013) | KC572203 | | 445 | 1634 - 2078 | 56 | 88 |
| Beebe N.W., et al (2013) | KC572204 | | 445 | 1634 - 2078 | 56 | 88 |
| Beebe N.W., et al (2013) | KC572205 | | 445 | 1634 - 2078 | 56 | 88 |
| Beebe N.W., et al (2013) | KC572206 | | 445 | 1634 - 2078 | 56 | 88 |
| Beebe N.W., et al (2013) | KC572207 | | 445 | 1634 - 2078 | 56 | 88 |
| Beebe N.W., et al (2013) | KC572208 | | 445 | 1634 - 2078 | 56 | 88 |
| Beebe N.W., et al (2013) | KC572209 | | 445 | 1634 - 2078 | 56 | 88 |
| Beebe N.W., et al (2013) | KC572210 | | 445 | 1634 - 2078 | 56 | 88 |
| Beebe N.W., et al (2013) | KC572211 | | 445 | 1634 - 2078 | 56 | 88 |
| Beebe N.W., et al (2013) | KC572212 | | 445 | 1634 - 2078 | 56 | 88 |
| Beebe N.W., et al (2013) | KC572213 | | 445 | 1634 - 2078 | 56 | 88 |
| Beebe N.W., et al (2013) | KC572214 | | 445 | 1634 - 2078 | 56 | 88 |
| Beebe N.W., et al (2013) | KC572215 | | 445 | 1634 - 2078 | 56 | 88 |
| Beebe N.W., et al (2013) | KC572216 | | 445 | 1634 - 2078 | 56 | 88 |
| Beebe N.W., et al (2013) | KC572217 | | 445 | 1634 - 2078 | 56 | 88 |
| Beebe N.W., et al (2013) | KC572218 | | 445 | 1634 - 2078 | 56 | 88 |
| Beebe N.W., et al (2013) | KC572219 | | 445 | 1634 - 2078 | 56 | 88 |
| Beebe N.W., et al (2013) | KC572220 | | 445 | 1634 - 2078 | 56 | 88 |
| Beebe N.W., et al (2013) | KC572221 | | 445 | 1634 - 2078 | 56 | 88 |
| Beebe N.W., et al (2013) | KC572222 | | 445 | 1634 - 2078 | 56 | 88 |
| Beebe N.W., et al (2013) | KC572223 | | 445 | 1634 - 2078 | 56 | 88 |
| Beebe N.W., et al (2013) | KC572224 | | 445 | 1634 - 2078 | 56 | 88 |
| Beebe N.W., et al (2013) | KC572225 | | 445 | 1634 - 2078 | 56 | 88 |
| Beebe N.W., et al (2013) | KC572226 | | 445 | 1634 - 2078 | 56 | 88 |
| Beebe N.W., et al (2013) | KC572227 | | 445 | 1634 - 2078 | 56 | 88 |
| Beebe N.W., et al (2013) | KC572228 | | 445 | 1634 - 2078 | 56 | 88 |
| Beebe N.W., et al (2013) | KC572229 | | 445 | 1634 - 2078 | 56 | 88 |
| Beebe N.W., et al (2013) | KC572230 | | 445 | 1634 - 2078 | 56 | 88 |
| Beebe N.W., et al (2013) | KC572231 | | 445 | 1634 - 2078 | 56 | 88 |
| Beebe N.W., et al (2013) | KC572232 | | 445 | 1634 - 2078 | 56 | 88 |
| Beebe N.W., et al (2013) | KC572233 | | 445 | 1634 - 2078 | 56 | 88 |
| Beebe N.W., et al (2013) | KC572234 | | 445 | 1634 - 2078 | 56 | 88 |
| Beebe N.W., et al (2013) | KC572235 | | 445 | 1634 - 2078 | 56 | 88 |
| Beebe N.W., et al (2013) | KC572236 | | 445 | 1634 - 2078 | 56 | 88 |
| Beebe N.W., et al (2013) | KC572237 | | 445 | 1634 - 2078 | 56 | 88 |
| Beebe N.W., et al (2013) | KC572238 | | 445 | 1634 - 2078 | 56 | 88 |
| Beebe N.W., et al (2013) | KC572239 | | 445 | 1634 - 2078 | 56 | 88 |
| Beebe N.W., et al (2013) | KC572240 | | 445 | 1634 - 2078 | 56 | 88 |
| Beebe N.W., et al (2013) | KC572241 | | 445 | 1634 - 2078 | 56 | 88 |
| Beebe N.W., et al (2013) | KC572242 | | 445 | 1634 - 2078 | 56 | 88 |
| Beebe N.W., et al (2013) | KC572243 | | 445 | 1634 - 2078 | 56 | 88 |
| Beebe N.W., et al (2013) | KC572244 | | 445 | 1634 - 2078 | 56 | 88 |
| Beebe N.W., et al (2013) | KC572245 | | 445 | 1634 - 2078 | 56 | 88 |
| Beebe N.W., et al (2013) | KC572246 | | 445 | 1634 - 2078 | 56 | 88 |
| Beebe N.W., et al (2013) | KC572247 | | 445 | 1634 - 2078 | 56 | 88 |
| Beebe N.W., et al (2013) | KC572248 | | 445 | 1634 - 2078 | 56 | 88 |
| Beebe N.W., et al (2013) | KC572249 | | 445 | 1634 - 2078 | 56 | 88 |
| Beebe N.W., et al (2013) | KC572250 | | 445 | 1634 - 2078 | 56 | 88 |
| Beebe N.W., et al (2013) | KC572251 | | 445 | 1634 - 2078 | 56 | 88 |
| Beebe N.W., et al (2013) | KC572252 | | 445 | 1634 - 2078 | 56 | 88 |
| Beebe N.W., et al (2013) | KC572253 | | 445 | 1634 - 2078 | 56 | 88 |
| Beebe N.W., et al (2013) | KC572254 | | 445 | 1634 - 2078 | 56 | 88 |
| Beebe N.W., et al (2013) | KC572255 | | 445 | 1634 - 2078 | 56 | 88 |
| Beebe N.W., et al (2013) | KC572256 | | 445 | 1634 - 2078 | 56 | 88 |
| Beebe N.W., et al (2013) | KC572257 | | 445 | 1634 - 2078 | 56 | 88 |
| Beebe N.W., et al (2013) | KC572258 | | 445 | 1634 - 2078 | 56 | 88 |
| Beebe N.W., et al (2013) | KC572259 | | 445 | 1634 - 2078 | 56 | 88 |
| Beebe N.W., et al (2013) | KC572260 | | 445 | 1634 - 2078 | 56 | 88 |
| Beebe N.W., et al (2013) | KC572261 | | 445 | 1634 - 2078 | 56 | 88 |
| Beebe N.W., et al (2013) | KC572262 | | 445 | 1634 - 2078 | 56 | 88 |
| Beebe N.W., et al (2013) | KC572263 | | 445 | 1634 - 2078 | 56 | 88 |
| Beebe N.W., et al (2013) | KC572264 | | 445 | 1634 - 2078 | 56 | 88 |
| Beebe N.W., et al (2013) | KC572265 | | 445 | 1634 - 2078 | 56 | 88 |
| Beebe N.W., et al (2013) | KC572266 | | 445 | 1634 - 2078 | 56 | 88 |
| Beebe N.W., et al (2013) | KC572267 | | 445 | 1634 - 2078 | 56 | 88 |
| Beebe N.W., et al (2013) | KC572268 | | 445 | 1634 - 2078 | 56 | 88 |
| Beebe N.W., et al (2013) | KC572269 | | 445 | 1634 - 2078 | 56 | 88 |
| Beebe N.W., et al (2013) | KC572270 | | 445 | 1634 - 2078 | 56 | 88 |
| Beebe N.W., et al (2013) | KC572271 | | 445 | 1634 - 2078 | 56 | 88 |
| Beebe N.W., et al (2013) | KC572272 | | 445 | 1634 - 2078 | 56 | 88 |
| Beebe N.W., et al (2013) | KC572273 | | 445 | 1634 - 2078 | 56 | 88 |
| Beebe N.W., et al (2013) | KC572274 | | 445 | 1634 - 2078 | 56 | 88 |
| Beebe N.W., et al (2013) | KC572275 | | 445 | 1634 - 2078 | 56 | 88 |
| Beebe N.W., et al (2013) | KC572276 | | 445 | 1634 - 2078 | 56 | 88 |
| Beebe N.W., et al (2013) | KC572277 | | 445 | 1634 - 2078 | 56 | 88 |
| Beebe N.W., et al (2013) | KC572278 | | 445 | 1634 - 2078 | 56 | 88 |
| Beebe N.W., et al (2013) | KC572279 | | 445 | 1634 - 2078 | 56 | 88 |
| Beebe N.W., et al (2013) | KC572280 | | 445 | 1634 - 2078 | 56 | 88 |
| Beebe N.W., et al (2013) | KC572281 | | 445 | 1634 - 2078 | 56 | 88 |
| Beebe N.W., et al (2013) | KC572282 | | 445 | 1634 - 2078 | 56 | 88 |
| Beebe N.W., et al (2013) | KC572283 | | 445 | 1634 - 2078 | 56 | 88 |
| Beebe N.W., et al (2013) | KC572284 | | 445 | 1634 - 2078 | 56 | 88 |
| Beebe N.W., et al (2013) | KC572285 | | 445 | 1634 - 2078 | 56 | 88 |
| Beebe N.W., et al (2013) | KC572286 | | 445 | 1634 - 2078 | 56 | 88 |
| Beebe N.W., et al (2013) | KC572287 | | 445 | 1634 - 2078 | 56 | 88 |
| Beebe N.W., et al (2013) | KC572288 | | 445 | 1634 - 2078 | 56 | 88 |
| Beebe N.W., et al (2013) | KC572289 | | 445 | 1634 - 2078 | 56 | 88 |
| Beebe N.W., et al (2013) | KC572290 | | 445 | 1634 - 2078 | 56 | 88 |
| Beebe N.W., et al (2013) | KC572291 | | 445 | 1634 - 2078 | 56 | 88 |
| Beebe N.W., et al (2013) | KC572292 | | 445 | 1634 - 2078 | 56 | 88 |
| Beebe N.W., et al (2013) | KC572293 | | 445 | 1634 - 2078 | 56 | 88 |
| Beebe N.W., et al (2013) | KC572294 | | 445 | 1634 - 2078 | 56 | 88 |
| Beebe N.W., et al (2013) | KC572295 | | 445 | 1634 - 2078 | 56 | 88 |
| Beebe N.W., et al (2013) | KC572296 | | 445 | 1634 - 2078 | 56 | 88 |
| Beebe N.W., et al (2013) | KC572297 | | 445 | 1634 - 2078 | 56 | 88 |
| Beebe N.W., et al (2013) | KC572298 | | 445 | 1634 - 2078 | 56 | 88 |
| Beebe N.W., et al (2013) | KC572299 | | 445 | 1634 - 2078 | 56 | 88 |
| Beebe N.W., et al (2013) | KC572300 | | 445 | 1634 - 2078 | 56 | 88 |
| Beebe N.W., et al (2013) | KC572301 | | 445 | 1634 - 2078 | 56 | 88 |
| Beebe N.W., et al (2013) | KC572302 | | 445 | 1634 - 2078 | 56 | 88 |
| Beebe N.W., et al (2013) | KC572303 | | 445 | 1634 - 2078 | 56 | 88 |
| Beebe N.W., et al (2013) | KC572304 | | 445 | 1634 - 2078 | 56 | 88 |
| Beebe N.W., et al (2013) | KC572305 | | 445 | 1634 - 2078 | 56 | 88 |
| Beebe N.W., et al (2013) | KC572306 | | 445 | 1634 - 2078 | 56 | 88 |
| Beebe N.W., et al (2013) | KC572307 | | 445 | 1634 - 2078 | 56 | 88 |
| Beebe N.W., et al (2013) | KC572308 | | 445 | 1634 - 2078 | 56 | 88 |
| Beebe N.W., et al (2013) | KC572309 | | 445 | 1634 - 2078 | 56 | 88 |
| Beebe N.W., et al (2013) | KC572310 | | 445 | 1634 - 2078 | 56 | 88 |
| Beebe N.W., et al (2013) | KC572311 | | 445 | 1634 - 2078 | 56 | 88 |
| Beebe N.W., et al (2013) | KC572312 | | 445 | 1634 - 2078 | 56 | 88 |
| Beebe N.W., et al (2013) | KC572313 | | 445 | 1634 - 2078 | 56 | 88 |
| Beebe N.W., et al (2013) | KC572314 | | 445 | 1634 - 2078 | 56 | 88 |
| Beebe N.W., et al (2013) | KC572315 | | 445 | 1634 - 2078 | 56 | 88 |
| Beebe N.W., et al (2013) | KC572316 | | 445 | 1634 - 2078 | 56 | 88 |
| Beebe N.W., et al (2013) | KC572317 | | 445 | 1634 - 2078 | 56 | 88 |
| Beebe N.W., et al (2013) | KC572318 | | 445 | 1634 - 2078 | 56 | 88 |
| Beebe N.W., et al (2013) | KC572319 | | 445 | 1634 - 2078 | 56 | 88 |
| Beebe N.W., et al (2013) | KC572320 | | 445 | 1634 - 2078 | 56 | 88 |
| Beebe N.W., et al (2013) | KC572321 | | 445 | 1634 - 2078 | 56 | 88 |
| Beebe N.W., et al (2013) | KC572322 | | 445 | 1634 - 2078 | 56 | 88 |
| Beebe N.W., et al (2013) | KC572323 | | 445 | 1634 - 2078 | 56 | 88 |
| Beebe N.W., et al (2013) | KC572324 | | 445 | 1634 - 2078 | 56 | 88 |
| Beebe N.W., et al (2013) | KC572325 | | 445 | 1634 - 2078 | 56 | 88 |
| Beebe N.W., et al (2013) | KC572326 | | 445 | 1634 - 2078 | 56 | 88 |
| Beebe N.W., et al (2013) | KC572327 | | 445 | 1634 - 2078 | 56 | 88 |
| Beebe N.W., et al (2013) | KC572328 | | 445 | 1634 - 2078 | 56 | 88 |
| Beebe N.W., et al (2013) | KC572329 | | 445 | 1634 - 2078 | 56 | 88 |
| Beebe N.W., et al (2013) | KC572330 | | 445 | 1634 - 2078 | 56 | 88 |
| Beebe N.W., et al (2013) | KC572331 | | 445 | 1634 - 2078 | 56 | 88 |
| Beebe N.W., et al (2013) | KC572332 | | 445 | 1634 - 2078 | 56 | 88 |
| Beebe N.W., et al (2013) | KC572333 | | 445 | 1634 - 2078 | 56 | 88 |
| Beebe N.W., et al (2013) | KC572334 | | 445 | 1634 - 2078 | 56 | 88 |
| Beebe N.W., et al (2013) | KC572335 | | 445 | 1634 - 2078 | 56 | 88 |
| Beebe N.W., et al (2013) | KC572336 | | 445 | 1634 - 2078 | 56 | 88 |
| Beebe N.W., et al (2013) | KC572337 | | 445 | 1634 - 2078 | 56 | 88 |
| Beebe N.W., et al (2013) | KC572338 | | 445 | 1634 - 2078 | 56 | 88 |
| Beebe N.W., et al (2013) | KC572339 | | 445 | 1634 - 2078 | 56 | 88 |
| Beebe N.W., et al (2013) | KC572340 | | 445 | 1634 - 2078 | 56 | 88 |
| Beebe N.W., et al (2013) | KC572341 | | 445 | 1634 - 2078 | 56 | 88 |
| Beebe N.W., et al (2013) | KC572342 | | 445 | 1634 - 2078 | 56 | 88 |
| Beebe N.W., et al (2013) | KC572343 | | 445 | 1634 - 2078 | 56 | 88 |
| Beebe N.W., et al (2013) | KC572344 | | 445 | 1634 - 2078 | 56 | 88 |
| Beebe N.W., et al (2013) | KC572345 | | 445 | 1634 - 2078 | 56 | 88 |
| Beebe N.W., et al (2013) | KC572346 | | 445 | 1634 - 2078 | 56 | 88 |
| Beebe N.W., et al (2013) | KC572347 | | 445 | 1634 - 2078 | 56 | 88 |
| Beebe N.W., et al (2013) | KC572348 | | 445 | 1634 - 2078 | 56 | 88 |
| Beebe N.W., et al (2013) | KC572349 | | 445 | 1634 - 2078 | 56 | 88 |
| Beebe N.W., et al (2013) | KC572350 | | 445 | 1634 - 2078 | 56 | 88 |
| Beebe N.W., et al (2013) | KC572351 | | 445 | 1634 - 2078 | 56 | 88 |
| Beebe N.W., et al (2013) | KC572352 | | 445 | 1634 - 2078 | 56 | 88 |
| Beebe N.W., et al (2013) | KC572353 | | 445 | 1634 - 2078 | 56 | 88 |
| Beebe N.W., et al (2013) | KC572354 | | 445 | 1634 - 2078 | 56 | 88 |
| Beebe N.W., et al (2013) | KC572355 | | 445 | 1634 - 2078 | 56 | 88 |
| Beebe N.W., et al (2013) | KC572356 | | 445 | 1634 - 2078 | 56 | 88 |
| Beebe N.W., et al (2013) | KC572357 | | 445 | 1634 - 2078 | 56 | 88 |
| Beebe N.W., et al (2013) | KC572358 | | 445 | 1634 - 2078 | 56 | 88 |
| Beebe N.W., et al (2013) | KC572359 | | 445 | 1634 - 2078 | 56 | 88 |
| Beebe N.W., et al (2013) | KC572360 | | 445 | 1634 - 2078 | 56 | 88 |
| Beebe N.W., et al (2013) | KC572361 | | 445 | 1634 - 2078 | 56 | 88 |
| Beebe N.W., et al (2013) | KC572362 | | 445 | 1634 - 2078 | 56 | 88 |
| Beebe N.W., et al (2013) | KC572363 | | 445 | 1634 - 2078 | 56 | 88 |
| Beebe N.W., et al (2013) | KC572364 | | 445 | 1634 - 2078 | 56 | 88 |
| Beebe N.W., et al (2013) | KC572365 | | 445 | 1634 - 2078 | 56 | 88 |
| Beebe N.W., et al (2013) | KC572366 | | 445 | 1634 - 2078 | 56 | 88 |
| Beebe N.W., et al (2013) | KC572367 | | 445 | 1634 - 2078 | 56 | 88 |
| Beebe N.W., et al (2013) | KC572368 | | 445 | 1634 - 2078 | 56 | 88 |
| Beebe N.W., et al (2013) | KC572369 | | 445 | 1634 - 2078 | 56 | 88 |
| Beebe N.W., et al (2013) | KC572370 | | 445 | 1634 - 2078 | 56 | 88 |
| Beebe N.W., et al (2013) | KC572371 | | 445 | 1634 - 2078 | 56 | 88 |
| Beebe N.W., et al (2013) | KC572372 | | 445 | 1634 - 2078 | 56 | 88 |
| Beebe N.W., et al (2013) | KC572373 | | 445 | 1634 - 2078 | 56 | 88 |
| Beebe N.W., et al (2013) | KC572374 | | 445 | 1634 - 2078 | 56 | 88 |
| Beebe N.W., et al (2013) | KC572375 | | 445 | 1634 - 2078 | 56 | 88 |
| Beebe N.W., et al (2013) | KC572376 | | 445 | 1634 - 2078 | 56 | 88 |
| Beebe N.W., et al (2013) | KC572377 | | 445 | 1634 - 2078 | 56 | 88 |
| Beebe N.W., et al (2013) | KC572378 | | 445 | 1634 - 2078 | 56 | 88 |
| Beebe N.W., et al (2013) | KC572379 | | 445 | 1634 - 2078 | 56 | 88 |
| Beebe N.W., et al (2013) | KC572380 | | 445 | 1634 - 2078 | 56 | 88 |
| Beebe N.W., et al (2013) | KC572381 | | 445 | 1634 - 2078 | 56 | 88 |
| Beebe N.W., et al (2013) | KC572382 | | 445 | 1634 - 2078 | 56 | 88 |
| Beebe N.W., et al (2013) | KC572383 | | 445 | 1634 - 2078 | 56 | 88 |
| Beebe N.W., et al (2013) | KC572384 | | 445 | 1634 - 2078 | 56 | 88 |
| Beebe N.W., et al (2013) | KC572385 | | 445 | 1634 - 2078 | 56 | 88 |
| Beebe N.W., et al (2013) | KC572386 | | 445 | 1634 - 2078 | 56 | 88 |
| Beebe N.W., et al (2013) | KC572387 | | 445 | 1634 - 2078 | 56 | 88 |
| Beebe N.W., et al (2013) | KC572388 | | 445 | 1634 - 2078 | 56 | 88 |
| Beebe N.W., et al (2013) | KC572389 | | 445 | 1634 - 2078 | 56 | 88 |
| Beebe N.W., et al (2013) | KC572390 | | 445 | 1634 - 2078 | 56 | 88 |
| Beebe N.W., et al (2013) | KC572391 | | 445 | 1634 - 2078 | 56 | 88 |
| Beebe N.W., et al (2013) | KC572392 | | 445 | 1634 - 2078 | 56 | 88 |
| Beebe N.W., et al (2013) | KC572393 | | 445 | 1634 - 2078 | 56 | 88 |
| Beebe N.W., et al (2013) | KC572394 | | 445 | 1634 - 2078 | 56 | 88 |
| Beebe N.W., et al (2013) | KC572395 | | 445 | 1634 - 2078 | 56 | 88 |
| Beebe N.W., et al (2013) | KC572396 | | 445 | 1634 - 2078 | 56 | 88 |
| Beebe N.W., et al (2013) | KC572397 | | 445 | 1634 - 2078 | 56 | 88 |
| Beebe N.W., et al (2013) | KC572398 | | 445 | 1634 - 2078 | 56 | 88 |
| Beebe N.W., et al (2013) | KC572399 | | 445 | 1634 - 2078 | 56 | 88 |
| Beebe N.W., et al (2013) | KC572400 | | 445 | 1634 - 2078 | 56 | 88 |
| Beebe N.W., et al (2013) | KC572401 | | 445 | 1634 - 2078 | 56 | 88 |
| Beebe N.W., et al (2013) | KC572402 | | 445 | 1634 - 2078 | 56 | 88 |
| Beebe N.W., et al (2013) | KC572403 | | 445 | 1634 - 2078 | 56 | 88 |
| Beebe N.W., et al (2013) | KC572404 | | 445 | 1634 - 2078 | 56 | 88 |
| Beebe N.W., et al (2013) | KC572405 | | 445 | 1634 - 2078 | 56 | 88 |
| Beebe N.W., et al (2013) | KC572406 | | 445 | 1634 - 2078 | 56 | 88 |
| Beebe N.W., et al (2013) | KC572407 | | 445 | 1634 - 2078 | 56 | 88 |
| Beebe N.W., et al (2013) | KC572408 | | 445 | 1634 - 2078 | 56 | 88 |
| Beebe N.W., et al (2013) | KC572409 | | 445 | 1634 - 2078 | 56 | 88 |
| Beebe N.W., et al (2013) | KC572410 | | 445 | 1634 - 2078 | 56 | 88 |
| Beebe N.W., et al (2013) | KC572411 | | 445 | 1634 - 2078 | 56 | 88 |
| Beebe N.W., et al (2013) | KC572412 | | 445 | 1634 - 2078 | 56 | 88 |
| Beebe N.W., et al (2013) | KC572413 | | 445 | 1634 - 2078 | 56 | 88 |
| Beebe N.W., et al (2013) | KC572414 | | 445 | 1634 - 2078 | 56 | 88 |
| Beebe N.W., et al (2013) | KC572415 | | 445 | 1634 - 2078 | 56 | 88 |
| Beebe N.W., et al (2013) | KC572416 | | 445 | 1634 - 2078 | 56 | 88 |
| Beebe N.W., et al (2013) | KC572417 | | 445 | 1634 - 2078 | 56 | 88 |
| Beebe N.W., et al (2013) | KC572418 | | 445 | 1634 - 2078 | 56 | 88 |
| Beebe N.W., et al (2013) | KC572419 | | 445 | 1634 - 2078 | 56 | 88 |
| Beebe N.W., et al (2013) | KC572420 | | 445 | 1634 - 2078 | 56 | 88 |
| Beebe N.W., et al (2013) | KC572421 | | 445 | 1634 - 2078 | 56 | 88 |
| Beebe N.W., et al (2013) | KC572422 | | 445 | 1634 - 2078 | 56 | 88 |
| Beebe N.W., et al (2013) | KC572423 | | 445 | 1634 - 2078 | 56 | 88 |
| Beebe N.W., et al (2013) | KC572424 | | 445 | 1634 - 2078 | 56 | 88 |
| Beebe N.W., et al (2013) | KC572425 | | 445 | 1634 - 2078 | 56 | 88 |
| Beebe N.W., et al (2013) | KC572426 | | 445 | 1634 - 2078 | 56 | 88 |
| Beebe N.W., et al (2013) | KC572427 | | 445 | 1634 - 2078 | 56 | 88 |
| Beebe N.W., et al (2013) | KC572428 | | 445 | 1634 - 2078 | 56 | 88 |
| Beebe N.W., et al (2013) | KC572429 | | 445 | 1634 - 2078 | 56 | 88 |
| Beebe N.W., et al (2013) | KC572430 | | 445 | 1634 - 2078 | 56 | 88 |
| Beebe N.W., et al (2013) | KC572431 | | 445 | 1634 - 2078 | 56 | 88 |
| Beebe N.W., et al (2013) | KC572432 | | 445 | 1634 - 2078 | 56 | 88 |
| Beebe N.W., et al (2013) | KC572433 | | 445 | 1634 - 2078 | 56 | 88 |
| Beebe N.W., et al (2013) | KC572434 | | 445 | 1634 - 2078 | 56 | 88 |
| Beebe N.W., et al (2013) | KC572435 | | 445 | 1634 - 2078 | 56 | 88 |
| Beebe N.W., et al (2013) | KC572436 | | 445 | 1634 - 2078 | 56 | 88 |
| Beebe N.W., et al (2013) | KC572437 | | 445 | 1634 - 2078 | 56 | 88 |
| Beebe N.W., et al (2013) | KC572438 | | 445 | 1634 - 2078 | 56 | 88 |
| Beebe N.W., et al (2013) | KC572439 | | 445 | 1634 - 2078 | 56 | 88 |
| Beebe N.W., et al (2013) | KC572440 | | 445 | 1634 - 2078 | 56 | 88 |
| Beebe N.W., et al (2013) | KC572441 | | 445 | 1634 - 2078 | 56 | 88 |
| Beebe N.W., et al (2013) | KC572442 | | 445 | 1634 - 2078 | 56 | 88 |
| Beebe N.W., et al (2013) | KC572443 | | 445 | 1634 - 2078 | 56 | 88 |
| Beebe N.W., et al (2013) | KC572444 | | 445 | 1634 - 2078 | 56 | 88 |
| Beebe N.W., et al (2013) | KC572445 | | 445 | 1634 - 2078 | 56 | 88 |
| Beebe N.W., et al (2013) | KC572446 | | 445 | 1634 - 2078 | 56 | 88 |
| Beebe N.W., et al (2013) | KC572447 | | 445 | 1634 - 2078 | 56 | 88 |
| Beebe N.W., et al (2013) | KC572448 | | 445 | 1634 - 2078 | 56 | 88 |
| Beebe N.W., et al (2013) | KC572449 | | 445 | 1634 - 2078 | 56 | 88 |
| Beebe N.W., et al (2013) | KC572450 | | 445 | 1634 - 2078 | 56 | 88 |
| Beebe N.W., et al (2013) | KC572451 | | 445 | 1634 - 2078 | 56 | 88 |
| Beebe N.W., et al (2013) | KC572452 | | 445 | 1634 - 2078 | 56 | 88 |
| Beebe N.W., et al (2013) | KC572453 | | 445 | 1634 - 2078 | 56 | 88 |
| Beebe N.W., et al (2013) | KC572454 | | 445 | 1634 - 2078 | 56 | 88 |
| Beebe N.W., et al (2013) | KC572455 | | 445 | 1634 - 2078 | 56 | 88 |
| Beebe N.W., et al (2013) | KC572456 | | 445 | 1634 - 2078 | 56 | 88 |
| Beebe N.W., et al (2013) | KC572457 | | 445 | 1634 - 2078 | 56 | 88 |
| Beebe N.W., et al (2013) | KC572458 | | 445 | 1634 - 2078 | 56 | 88 |
| Beebe N.W., et al (2013) | KC572459 | | 445 | 1634 - 2078 | 56 | 88 |
| Beebe N.W., et al (2013) | KC572460 | | 445 | 1634 - 2078 | 56 | 88 |
| Beebe N.W., et al (2013) | KC572461 | | 445 | 1634 - 2078 | 56 | 88 |
| Beebe N.W., et al (2013) | KC572462 | | 445 | 1634 - 2078 | 56 | 88 |
| Beebe N.W., et al (2013) | KC572463 | | 445 | 1634 - 2078 | 56 | 88 |
| Beebe N.W., et al (2013) | KC572464 | | 445 | 1634 - 2078 | 56 | 88 |
| Beebe N.W., et al (2013) | KC572465 | | 445 | 1634 - 2078 | 56 | 88 |
| Beebe N.W., et al (2013) | KC572466 | | 445 | 1634 - 2078 | 56 | 88 |
| Beebe N.W., et al (2013) | KC572467 | | 445 | 1634 - 2078 | 56 | 88 |
| Beebe N.W., et al (2013) | KC572468 | | 445 | 1634 - 2078 | 56 | 88 |
| Beebe N.W., et al (2013) | KC572469 | | 445 | 1634 - 2078 | 56 | 88 |
| Beebe N.W., et al (2013) | KC572470 | | 445 | 1634 - 2078 | 56 | 88 |
| Beebe N.W., et al (2013) | KC572471 | | 445 | 1634 - 2078 | 56 | 88 |
| Beebe N.W., et al (2013) | KC572472 | | 445 | 1634 - 2078 | 56 | 88 |
| Beebe N.W., et al (2013) | KC572473 | | 445 | 1634 - 2078 | 56 | 88 |
| Beebe N.W., et al (2013) | KC572474 | | 445 | 1634 - 2078 | 56 | 88 |
| Beebe N.W., et al (2013) | KC572475 | | 445 | 1634 - 2078 | 56 | 88 |
| Beebe N.W., et al (2013) | KC572476 | | 445 | 1634 - 2078 | 56 | 88 |
| Beebe N.W., et al (2013) | KC572477 | | 445 | 1634 - 2078 | 56 | 88 |
| Beebe N.W., et al (2013) | KC572478 | | 445 | 1634 - 2078 | 56 | 88 |
| Beebe N.W., et al (2013) | KC572479 | | 445 | 1634 - 2078 | 56 | 88 |
| Beebe N.W., et al (2013) | KC572480 | | 445 | 1634 - 2078 | 56 | 88 |
| Beebe N.W., et al (2013) | KC572481 | | 445 | 1634 - 2078 | 56 | 88 |
| Beebe N.W., et al (2013) | KC572482 | | 445 | 1634 - 2078 | 56 | 88 |
| Beebe N.W., et al (2013) | KC572483 | | 445 | 1634 - 2078 | 56 | 88 |
| Beebe N.W., et al (2013) | KC572484 | | 445 | 1634 - 2078 | 56 | 88 |
| Beebe N.W., et al (2013) | KC572485 | | 445 | 1634 - 2078 | 56 | 88 |
| Beebe N.W., et al (2013) | KC572486 | | 445 | 1634 - 2078 | 56 | 88 |
| Beebe N.W., et al (2013) | KC572487 | | 445 | 1634 - 2078 | 56 | 88 |
| Beebe N.W., et al (2013) | KC572488 | | 445 | 1634 - 2078 | 56 | 88 |
| Beebe N.W., et al (2013) | KC572489 | | 445 | 1634 - 2078 | 56 | 88 |
| Beebe N.W., et al (2013) | KC572490 | | 445 | 1634 - 2078 | 56 | 88 |
| Beebe N.W., et al (2013) | KC572491 | | 445 | 1634 - 2078 | 56 | 88 |
| Beebe N.W., et al (2013) | KC572492 | | 445 | 1634 - 2078 | 56 | 88 |
| Beebe N.W., et al (2013) | KC572493 | | 445 | 1634 - 2078 | 56 | 88 |
| Beebe N.W., et al (2013) | KC572494 | | 445 | 1634 - 2078 | 56 | 88 |
| Beebe N.W., et al (2013) | KC572495 | | 445 | 1634 - 2078 | 56 | 88 |
| Beebe N.W., et al (2013) | KC572496 | | 445 | 1634 - 2078 | 56 | 88 |
| Raharimalala F.N., et al (2012) | JN406724 | | 573 | 1581 - 2170 | 0 | 100 |
| Raharimalala F.N., et al (2012) | JN406725 | | 573 | 1581 - 2170 | 0 | 100 |
| Raharimalala F.N., et al (2012) | JN406726 | | 573 | 1581 - 2170 | 0 | 100 |
| Raharimalala F.N., et al (2012) | JN406729 | | 573 | 1581 - 2170 | 0 | 100 |
| **Raharimalala F.N., et al (2012)** | **†JN406659** | | **573** | **1581 - 2170** | **0** | **100** |
| Raharimalala F.N., et al (2012) | JN406660 | | 573 | 1581 - 2170 | 0 | 100 |
| Raharimalala F.N., et al (2012) | JN406661 | | 573 | 1581 - 2170 | 0 | 100 |
| Raharimalala F.N., et al (2012) | JN406676 | | 573 | 1581 - 2170 | 0 | 100 |
| Raharimalala F.N., et al (2012) | JN406688 | | 573 | 1581 - 2170 | 0 | 100 |
| Raharimalala F.N., et al (2012) | JN406696 | | 573 | 1581 - 2170 | 0 | 100 |
| Raharimalala F.N., et al (2012) | JN406699 | | 573 | 1581 - 2170 | 0 | 100 |
| Porretta D., et al. (2012) | JQ436981 | | 689 | 2282 - 2970 | 461 | 0 |
| Porretta D., et al. (2012) | JQ436982 | | 689 | 2282 - 2970 | 461 | 0 |
| Porretta D., et al. (2012) | JQ436983 | | 689 | 2282 - 2970 | 461 | 0 |
| Porretta D., et al. (2012) | JQ436984 | | 689 | 2282 - 2970 | 461 | 0 |
| Porretta D., et al. (2012) | JQ436985 | | 689 | 2282 - 2970 | 461 | 0 |
| Porretta D., et al. (2012) | JQ436986 | | 689 | 2282 - 2970 | 461 | 0 |
| Porretta D., et al. (2012) | JQ436987 | | 689 | 2282 - 2970 | 461 | 0 |
| Porretta D., et al. (2012) | JQ436988 | | 689 | 2282 - 2970 | 461 | 0 |
| Porretta D., et al. (2012) | JQ436989 | | 689 | 2282 - 2970 | 461 | 0 |
| Porretta D., et al. (2012) | JQ436990 | | 689 | 2282 - 2970 | 461 | 0 |
| Porretta D., et al. (2012) | JQ436991 | | 689 | 2282 - 2970 | 461 | 0 |
| Porretta D., et al. (2012) | JQ436992 | | 689 | 2282 - 2970 | 461 | 0 |
| Porretta D., et al. (2012) | JQ436993 | | 689 | 2282 - 2970 | 461 | 0 |
| Porretta D., et al. (2012) | JQ436994 | | 689 | 2282 - 2970 | 461 | 0 |
| Porretta D., et al. (2012) | JQ436995 | | 689 | 2282 - 2970 | 461 | 0 |
| Porretta D., et al. (2012) | JQ436996 | | 689 | 2282 - 2970 | 461 | 0 |
| Porretta D., et al. (2012) | JQ436997 | | 689 | 2282 - 2970 | 461 | 0 |
| Porretta D., et al. (2012) | JQ436998 | | 689 | 2282 - 2970 | 461 | 0 |
| Porretta D., et al. (2012) | JQ436999 | | 689 | 2282 - 2970 | 461 | 0 |
| Porretta D., et al. (2012) | JQ437000 | | 689 | 2282 - 2970 | 461 | 0 |
| Porretta D., et al. (2012) | JQ437001 | | 689 | 2282 - 2970 | 461 | 0 |
| Porretta D., et al. (2012) | JQ437002 | | 689 | 2282 - 2970 | 461 | 0 |
| Porretta D., et al. (2012) | JQ437003 | | 689 | 2282 - 2970 | 461 | 0 |
| Porretta D., et al. (2012) | JQ437004 | | 689 | 2282 - 2970 | 461 | 0 |
| Porretta D., et al. (2012) | JQ437005 | | 689 | 2282 - 2970 | 461 | 0 |
| Porretta D., et al. (2012) | JQ437006 | | 689 | 2282 - 2970 | 461 | 0 |
| Porretta D., et al. (2012) | JQ437007 | | 689 | 2282 - 2970 | 461 | 0 |
| Porretta D., et al. (2012) | JQ437008 | | 689 | 2282 - 2970 | 461 | 0 |
| Kamgang B., et al (2011) | JF309317 | | 426 | 1702 - 2127 | 35 | 92 |
| Kamgang B., et al (2011) | JF309318 | | 426 | 1702 - 2127 | 35 | 92 |
| Kamgang B., et al (2011) | JF309319 | | 426 | 1702 - 2127 | 35 | 92 |
| Kamgang B., et al (2011) | JF309320 | | 426 | 1702 - 2127 | 35 | 92 |
| Kamgang B., et al (2013) | KC979140 | | 426 | 1702 - 2127 | 35 | 92 |
| Kamgang B., et al (2011) | KC979141 | | 426 | 1702 - 2127 | 35 | 92 |
| Kamgang B., et al (2011) | KC979142 | | 426 | 1702 - 2127 | 35 | 92 |
| Kamgang B., et al (2011) | KC979143 | | 426 | 1702 - 2127 | 35 | 92 |
| Delatte H., et al (2011) | HQ622904 | | 452 | 1674 - 2122 | 9 | 98 |
| Delatte H., et al (2011) | HQ622905 | | 452 | 1674 - 2122 | 9 | 98 |
| Delatte H., et al (2011) | HQ622906 | | 452 | 1674 - 2122 | 9 | 98 |
| Delatte H., et al (2011) | HQ622907 | | 452 | 1674 - 2122 | 9 | 98 |
| Delatte H., et al (2011) | HQ622908 | | 452 | 1674 - 2122 | 9 | 98 |
| Delatte H., et al (2011) | HQ622909 | | 452 | 1674 - 2122 | 9 | 98 |
| Delatte H., et al (2011) | HQ622910 | | 452 | 1674 - 2122 | 9 | 98 |
| Delatte H., et al (2011) | HQ622911 | | 452 | 1674 - 2122 | 9 | 98 |
| Delatte H., et al (2011) | HQ622912 | | 452 | 1674 - 2122 | 9 | 98 |
| Delatte H., et al (2011) | HQ622913 | | 452 | 1674 - 2122 | 9 | 98 |
| Delatte H., et al (2011) | HQ622914 | | 452 | 1674 - 2122 | 9 | 98 |
| Delatte H., et al (2011) | HQ622915 | | 452 | 1674 - 2122 | 9 | 98 |
| Delatte H., et al (2011) | HQ622916 | | 452 | 1674 - 2122 | 9 | 98 |
| Delatte H., et al (2011) | HQ622917 | | 452 | 1674 - 2122 | 9 | 98 |
| Delatte H., et al (2011) | HQ622918 | | 452 | 1674 - 2122 | 9 | 98 |
| Delatte H., et al (2011) | HQ622919 | | 452 | 1674 - 2122 | 9 | 98 |
| Delatte H., et al (2011) | HQ622920 | | 452 | 1674 - 2122 | 9 | 98 |
| Delatte H., et al (2011) | HQ622921 | | 452 | 1674 - 2122 | 9 | 98 |
| Delatte H., et al (2011) | HQ622922 | | 452 | 1674 - 2122 | 9 | 98 |
| Delatte H., et al (2011) | HQ622923 | | 452 | 1674 - 2122 | 9 | 98 |
| Delatte H., et al (2011) | HQ622924 | | 452 | 1674 - 2122 | 9 | 98 |
| Delatte H., et al (2011) | HQ622925 | | 452 | 1674 - 2122 | 9 | 98 |
| Delatte H., et al (2011) | HQ622926 | | 452 | 1674 - 2122 | 9 | 98 |
| Delatte H., et al (2011) | HQ622927 | | 452 | 1674 - 2122 | 9 | 98 |
| Delatte H., et al (2011) | HQ622928 | | 452 | 1674 - 2122 | 9 | 98 |
| Delatte H., et al (2011) | HQ622929 | | 452 | 1674 - 2122 | 9 | 98 |
| Delatte H., et al (2011) | HQ622930 | | 452 | 1674 - 2122 | 9 | 98 |
| Delatte H., et al (2011) | HQ622931 | | 452 | 1674 - 2122 | 9 | 98 |
| Delatte H., et al (2011) | HQ622932 | | 452 | 1674 - 2122 | 9 | 98 |
| Delatte H., et al (2011) | HQ622933 | | 452 | 1674 - 2122 | 9 | 98 |
| Delatte H., et al (2011) | HQ622934 | | 452 | 1674 - 2122 | 9 | 98 |
| Delatte H., et al (2011) | HQ622935 | | 452 | 1674 - 2122 | 9 | 98 |
| Delatte H., et al (2011) | HQ622936 | | 452 | 1674 - 2122 | 9 | 98 |
| Delatte H., et al (2011) | HQ622937 | | 452 | 1674 - 2122 | 9 | 98 |
| Delatte H., et al (2011) | HQ622938 | | 452 | 1674 - 2122 | 9 | 98 |
| Delatte H., et al (2011) | HQ622938 | | 452 | 1674 - 2122 | 9 | 98 |
| Delatte H., et al (2011) | HQ622940 | | 452 | 1674 - 2122 | 9 | 98 |
| Delatte H., et al (2011) | HQ622941 | | 452 | 1674 - 2122 | 9 | 98 |
| Delatte H., et al (2011) | HQ622942 | | 452 | 1674 - 2122 | 9 | 98 |
| Delatte H., et al (2011) | HQ622943 | | 452 | 1674 - 2122 | 9 | 98 |
| Delatte H., et al (2011) | HQ622944 | | 452 | 1674 - 2122 | 9 | 98 |
| Delatte H., et al (2011) | HQ622945 | | 452 | 1674 - 2122 | 9 | 98 |
| Delatte H., et al (2011) | HQ622946 | | 452 | 1674 - 2122 | 9 | 98 |
| Delatte H., et al (2011) | HQ622947 | | 452 | 1674 - 2122 | 9 | 98 |
| Delatte H., et al (2011) | HQ622948 | | 452 | 1674 - 2122 | 9 | 98 |
| Delatte H., et al (2011) | HQ622949 | | 452 | 1674 - 2122 | 9 | 98 |
| Delatte H., et al (2011) | HQ622950 | | 452 | 1674 - 2122 | 9 | 98 |
| Delatte H., et al (2011) | HQ622951 | | 452 | 1674 - 2122 | 9 | 98 |
| Delatte H., et al (2011) | HQ622952 | | 452 | 1674 - 2122 | 9 | 98 |
| Delatte H., et al (2011) | HQ622953 | | 452 | 1674 - 2122 | 9 | 98 |
| Delatte H., et al (2011) | HQ622954 | | 452 | 1674 - 2122 | 9 | 98 |
| Delatte H., et al (2011) | HQ622955 | | 452 | 1674 - 2122 | 9 | 98 |
| Delatte H., et al (2011) | HQ622956 | | 452 | 1674 - 2122 | 9 | 98 |
| Delatte H., et al (2011) | HQ622957 | | 452 | 1674 - 2122 | 9 | 98 |
| Delatte H., et al (2011) | HQ622958 | | 452 | 1674 - 2122 | 9 | 98 |
| Delatte H., et al (2011) | HQ622959 | | 452 | 1674 - 2122 | 9 | 98 |
| Delatte H., et al (2011) | HQ622960 | | 452 | 1674 - 2122 | 9 | 98 |
| Delatte H., et al (2011) | HQ622961 | | 452 | 1674 - 2122 | 9 | 98 |
| Delatte H., et al (2011) | HQ622962 | | 452 | 1674 - 2122 | 9 | 98 |
| Delatte H., et al (2011) | HQ622963 | | 452 | 1674 - 2122 | 9 | 98 |
| Delatte H., et al (2011) | HQ622964 | | 452 | 1674 - 2122 | 9 | 98 |
| Delatte H., et al (2011) | HQ622965 | | 452 | 1674 - 2122 | 9 | 98 |
| Delatte H., et al (2011) | HQ622966 | | 452 | 1674 - 2122 | 9 | 98 |
| Delatte H., et al (2011) | HQ622967 | | 452 | 1674 - 2122 | 9 | 98 |
| Delatte H., et al (2011) | HQ622968 | | 452 | 1674 - 2122 | 9 | 98 |
| Delatte H., et al (2011) | HQ622969 | | 452 | 1674 - 2122 | 9 | 98 |
| Delatte H., et al (2011) | HQ622970 | | 452 | 1674 - 2122 | 9 | 98 |
| Delatte H., et al (2011) | HQ622971 | | 452 | 1674 - 2122 | 9 | 98 |
| Delatte H., et al (2011) | HQ622972 | | 452 | 1674 - 2122 | 9 | 98 |
| Delatte H., et al (2011) | HQ622973 | | 452 | 1674 - 2122 | 9 | 98 |
| Delatte H., et al (2011) | HQ622974 | | 452 | 1674 - 2122 | 9 | 98 |
| Delatte H., et al (2011) | HQ622975 | | 452 | 1674 - 2122 | 9 | 98 |
| Delatte H., et al (2011) | HQ622976 | | 452 | 1674 - 2122 | 9 | 98 |
| Delatte H., et al (2011) | HQ622977 | | 452 | 1674 - 2122 | 9 | 98 |
| Delatte H., et al (2011) | HQ622978 | | 452 | 1674 - 2122 | 9 | 98 |
| Delatte H., et al (2011) | HQ622979 | | 452 | 1674 - 2122 | 9 | 98 |
| Delatte H., et al (2011) | HQ622980 | | 452 | 1674 - 2122 | 9 | 98 |
| Delatte H., et al (2011) | HQ622981 | | 452 | 1674 - 2122 | 9 | 98 |
| Delatte H., et al (2011) | HQ622982 | | 452 | 1674 - 2122 | 9 | 98 |
| Delatte H., et al (2011) | HQ622983 | | 452 | 1674 - 2122 | 9 | 98 |
| Delatte H., et al (2011) | HQ622984 | | 452 | 1674 - 2122 | 9 | 98 |
| Delatte H., et al (2011) | *HQ622985 | | 452 | 1674 - 2122 | 9 | 98 |
| Delatte H., et al (2011) | HQ622986 | | 452 | 1674 - 2122 | 9 | 98 |
| Delatte H., et al (2011) | HQ622987 | | 452 | 1674 - 2122 | 9 | 98 |
| Delatte H., et al (2011) | HQ622988 | | 452 | 1674 - 2122 | 9 | 98 |
| Delatte H., et al (2011) | HQ622989 | | 452 | 1674 - 2122 | 9 | 98 |
| Delatte H., et al (2011) | HQ622990 | | 452 | 1674 - 2122 | 9 | 98 |
| Delatte H., et al (2011) | HQ622991 | | 452 | 1674 - 2122 | 9 | 98 |
| Delatte H., et al (2011) | HQ622992 | | 452 | 1674 - 2122 | 9 | 98 |
| Delatte H., et al (2011) | HQ622993 | | 452 | 1674 - 2122 | 9 | 98 |
| Delatte H., et al (2011) | HQ622994 | | 452 | 1674 - 2122 | 9 | 98 |
| Delatte H., et al (2011) | HQ622995 | | 452 | 1674 - 2122 | 9 | 98 |
| Delatte H., et al (2011) | HQ622996 | | 452 | 1674 - 2122 | 9 | 98 |
| Delatte H., et al (2011) | HQ622997 | | 452 | 1674 - 2122 | 9 | 98 |
| Delatte H., et al (2011) | HQ622998 | | 452 | 1674 - 2122 | 9 | 98 |
| Delatte H., et al (2011) | HQ622999 | | 452 | 1674 - 2122 | 9 | 98 |
| Delatte H., et al (2011) | HQ623000 | | 452 | 1674 - 2122 | 9 | 98 |
| Delatte H., et al (2011) | HQ623001 | | 452 | 1674 - 2122 | 9 | 98 |
| Delatte H., et al (2011) | HQ623002 | | 452 | 1674 - 2122 | 9 | 98 |
| Delatte H., et al (2011) | HQ623003 | | 452 | 1674 - 2122 | 9 | 98 |
| Delatte H., et al (2011) | HQ623004 | | 452 | 1674 - 2122 | 9 | 98 |
| Delatte H., et al (2011) | HQ623005 | | 452 | 1674 - 2122 | 9 | 98 |
| Delatte H., et al (2011) | HQ623006 | | 452 | 1674 - 2122 | 9 | 98 |
| **Eskildsen et al. *in revision*** | | **KX171395** | **461** | **1674 - 2131** | **0** | **100** |
| **Eskildsen et al. *in revision*** | | **KX171396** | **461** | **1674 - 2131** | **0** | **100** |
| **Eskildsen et al. *in revision*** | | **KX171397** | **461** | **1674 - 2131** | **0** | **100** |
| **Eskildsen et al. *in revision*** | | **KX171398** | **461** | **1674 - 2131** | **0** | **100** |
| **Eskildsen et al. *in revision*** | | **KX171399** | **461** | **1674 - 2131** | **0** | **100** |
| **Eskildsen et al. *in revision*** | | **KX171400** | **461** | **1674 - 2131** | **0** | **100** |
| **Eskildsen et al. *in revision*** | | **KX171401** | **461** | **1674 - 2131** | **0** | **100** |
| **Eskildsen et al. *in revision*** | | **KX171402** | **461** | **1674 - 2131** | **0** | **100** |

**Methodology (Sorting process)**:

We searched for *CO1* gene DNA sequences of *Aedes albopictus* from publications suggested by Reviewers (S4 Table) using bioinformatics tools at the NCBI webpage. We then selected those with a similarity score of 99% or above, which overlapped with the exact position and length (100%) of our Panamanian *CO1* gene sequences (Genbank accession numbers KX171395 – KX171402 in Table 2). See the step-by-step procedure below; also see S4 Table and Figure A for more details about the analysis conducted here and the findings we got.

DNA sequences from Vincenza Battaglia et al. (2016) [14 sequences]; Zhang et al. (2015) [1 sequence]; Shaikevich et al. (2013) [2 sequences] and Raharimalala et al. (2012) [1 sequence] fulfilled our sorting criteria and were therefore included in further phylogenetic analysis. Vincenza Battaglia et al. (2016) included full mitochondrial genome sequences (Total length of 13,792 base pairs) of *Aedes albopictus* from three different continents, including the Americas, Asia and Europe.

**Procedure**:

1. First, we located mitogenome sequences from *Aedes albopictus* using the nucleotide search tool at NCBI and the corresponding accession numbers in Vincenza Battaglia et al. (2016). We did the same with *CO1* sequences from other papers/authors.

2. Second, we used Graphics at NCBI (e.g., *Aedes albopictus* isolate 9#Bra mitochondrion, partial genome) to locate the CDS ID (e.g., APC25272.1) of the *CO1* gene (e.g., Location in the mitogenome = 1,116– 2,625 pb; Span = 1,537 pb; Product = 512) within each mitogenome, and FASTA view to obtain the fasta version of the entire *CO1* gene sequence. We repeated this procedure 26 times to obtain all the *CO1* gene sequences from the mitogenomes reported in Vincenza Battaglia et al. (2016).

3. We then coalesced full *CO1* gene DNA sequences from Vincenza Battaglia et al. (2016) into unique sequences (e.g., Haplotypes) and selected between 1 and 2 representative haplotype members of each Haplogroups (A1, A2 and A3 from Figure 1 of that publication “Phylogeny of *Ae. albopictus* mitogenomes”).

4. We then compared these sequences with our partial *CO1* gene sequences to find the piece that overlap exactly. Complete *CO1* gene sequences in FASTA format from Vincenza Battaglia et al. (2016) were BLASTed against all the Panamanian *CO1* sequences to obtain an initial alignment. We did the same with *CO1* sequences from other papers.

5. This initial alignment was then imported into MEGA version 7.0 and sequences were re-aligned using a pairwise multiple alignment in ClustalW with default parameters.

6. All the sequences were then cut to a similar size matching the length of Panamanian sequences in S4 Table.

7. We run a NJ phylogenetic tree in MEGA version 7.0, with Kimura 2 parameters as a model of evolution and 1000 bootstrap replicates (Figure A). This NJ phylogenetic tree was built using the exact same setting as in the previous version of our manuscript.

8. See the interpretation of these results with regards to the suggestion by Reviewers, right after Figure A.

**Figure A**. **(To the left)** NJ phylogenetic tree constructed in MEGA 7.0 using *Aedes albopictus* *CO1* gene haplotypes from Panama (Eskildsen et al. *in revision*) [*8 sequences represented by Black triangles*]; plus sequences from Vincenza Battaglia et al. (2016) [*14 sequences represented by green squares*]; Zhang et al. (2015) [*1 sequence represented by green square*]; Shaikevich et al. (2013) [*2 sequences represented by red squares*] and Raharimalala et al. (2012) [*1 sequence represented by red square*]. **(To the right)** Original NJ phylogenetic tree from Eskildsen et al., in revision in Plos One.

**Explanation**: The new topology (Located to the left in Figure A) matched almost exactly the one from our previous Phylogenetic analysis (Located to the right in Figure A). In addition, the position of the Panamanian haplotypes of *Aedes albopictus* continued to be the same as in our original Phylogenetic tree. Therefore, this new analysis strengthens even more our initial conclusions about the potential Geographic origin and distribution of invading *Aedes albopictus* in Panama. Most mitogenome *CO1* gene sequences clustered together with members of sub-clade B(b), confirming the widespread distribution of this maternal lineage of *Aedes albopictus*. However, they did not contribute much information to sub-clade B(c) and sub-clade B(a). Lastly, not a single additional sequence from these studies clustered together with members of Clade A, which is the most prominent in Panama.
