# Supplementary figures and images for: Maternal invasion history of Aedes aegypti and Aedes albopictus into the Isthmus of Panama: Implications for the control of emergent viral disease agents
Source: PLoS One. 2018 Mar 26;13(3):e0194874. doi: 10.1371/journal.pone.0194874 (PMC5868824; doi:10.1371/journal.pone.0194874)

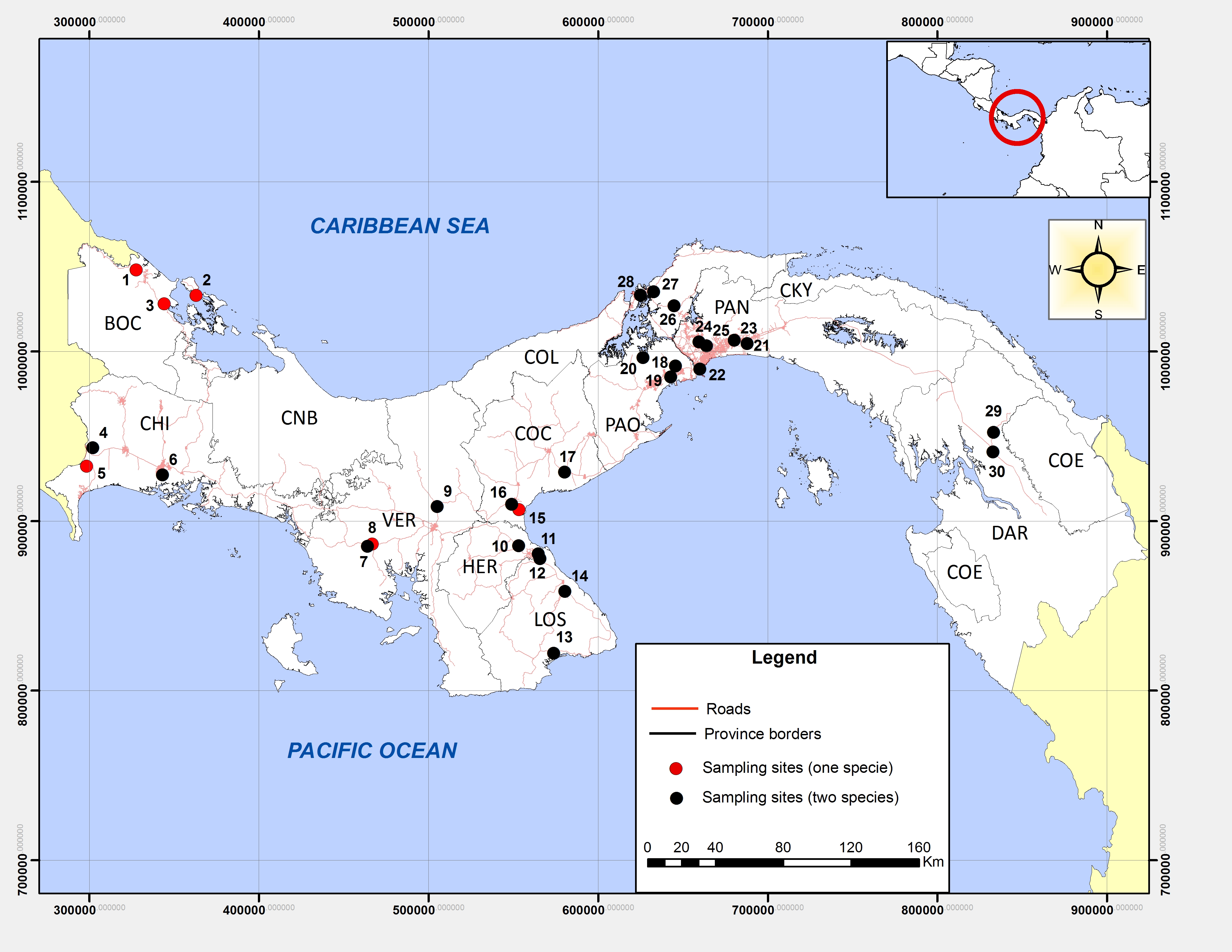

Supplement: S1 Fig — Circles represent the locations of each locality. Black and red circles represent the presence of both or only one species. The numbers represent the locality in Table 1. (TIFF) [file pone.0194874.s005.tiff]

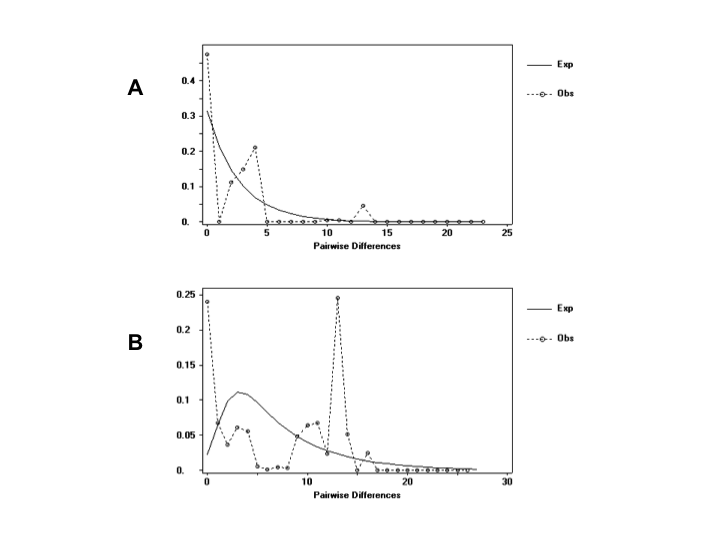

Supplement: S2 Fig — Mismatch distribution of CO1 sequences of Aedes mosquitoes of Panama (A: Ae. aegypti and B: Ae. albopictus). 122 sequences comprising 728 base pairs for Aedes aegypti and 117 sequences comprising 461 bp from Aedes albopictus were used in these analyses. (TIFF) [file pone.0194874.s006.tiff]

## Slide 1
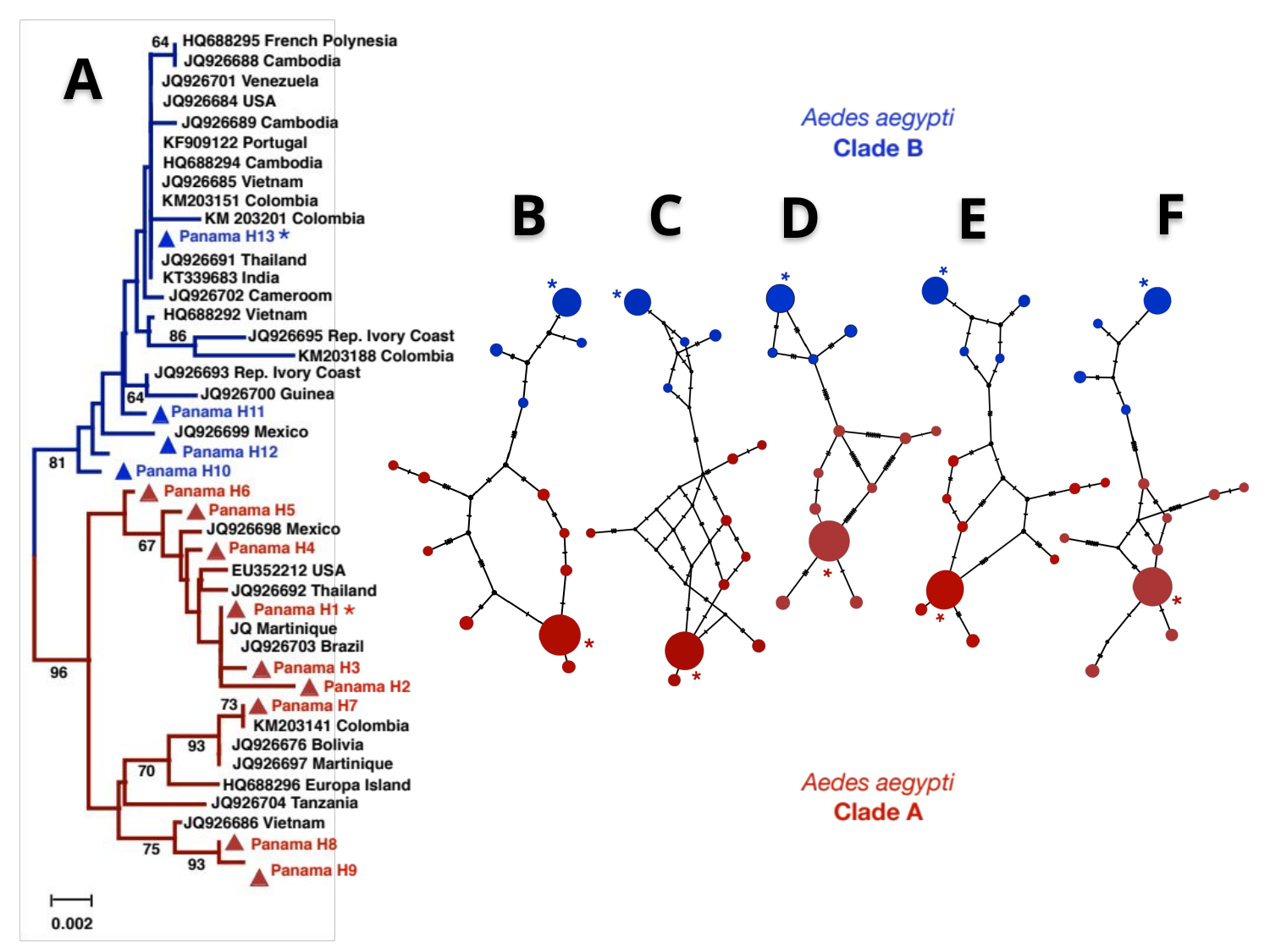

A
F
B
C
D
E

Supplement: S3 Fig — (A) Neighbor-joining phylogenetic tree of Panamanian and worldwide CO1 haplotypes of Aedes aegypti from GenBank (http://blast.ncbi.nlm.nih.gov/). Panamanian haplotypes belonging to Clade A and Clade B are shown in red and blue triangles, respectively. Bootstrap values depicting branch support higher than 60% are shown in the tree. Asterisk in Haplotype 1 and Haplotype 13 indicate most frequent Panamanian variants within Clade A and B, in that order. (B–F / left to right) Haplotype relationships of Ae. aegypti given by The Minimum Spanning (MS), Median Joining (MJ), Tight Span Walker (TSW), Integer NJ Net (INJN) and Parsimony (TCS) networks, respectively. (PPTX) [file pone.0194874.s007.pptx]

## Slide 1
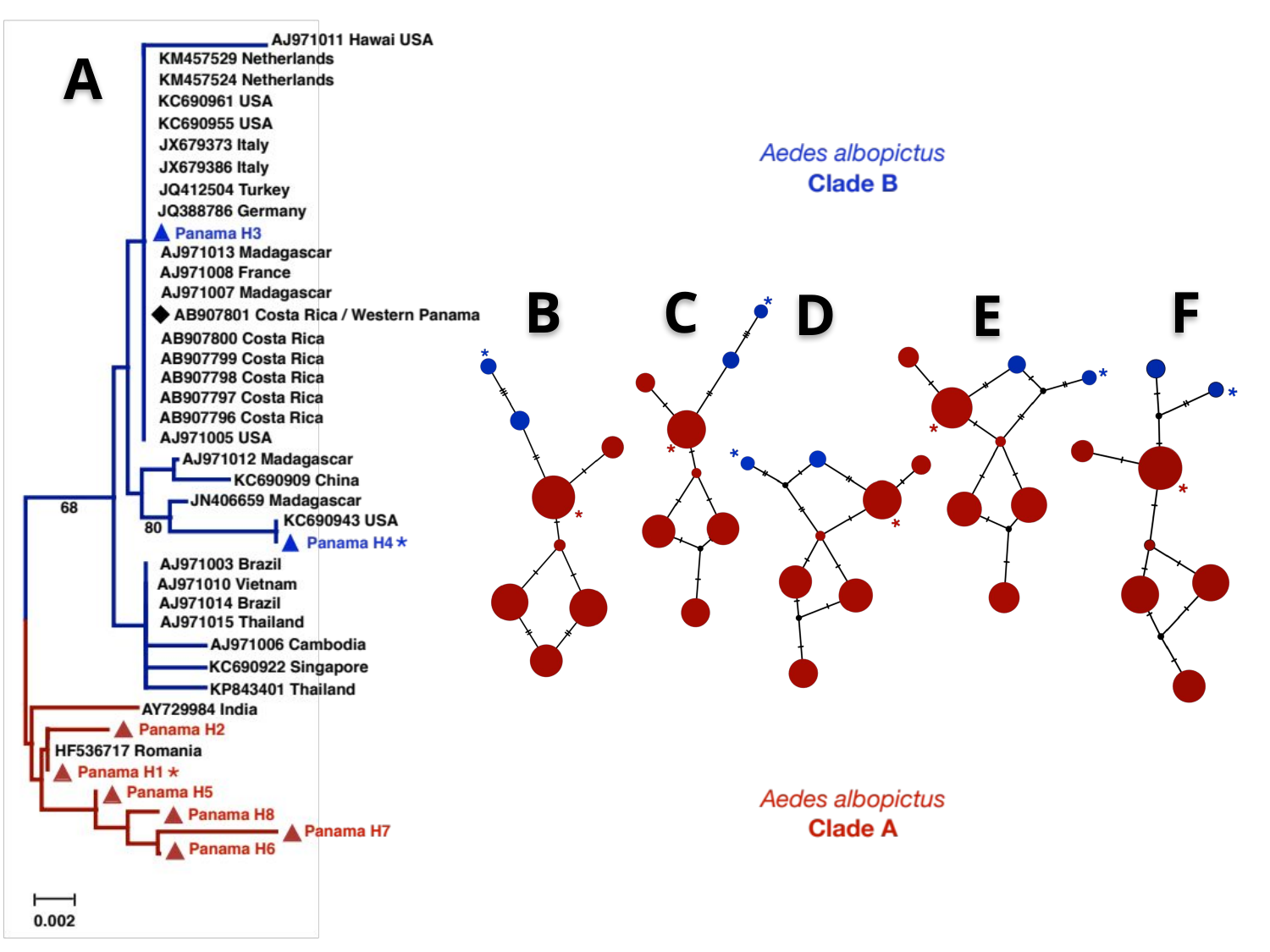

A
F
B
C
D
E

Supplement: S4 Fig — (A) Neighbor-joining phylogenetic tree of Panamanian and worldwide CO1 haplotypes of Aedes albopictus from GenBank (http://blast.ncbi.nlm.nih.gov/). Panamanian haplotypes belonging to Clade A and Clade B are shown in red and blue triangles, respectively. Bootstrap values depicting branch support higher than 60% are shown in the tree. Asterisks (*) in Haplotype 1 and Haplotype 4 indicate most frequent variants in Clade A and B, respectively. (B–F / left to right) Haplotype relationships of Ae. albopictus given by The Minimum Spanning (MS), Median Joining (MJ), Tight Span Walker (TSW), Integer NJ Net (INJN) and Parsimony (TCS) networks, respectively. (PPTX) [file pone.0194874.s008.pptx]
